# Vat Photopolymerization Printing of Modular Soft Stretchable Low-Cost Elastomers

**DOI:** 10.1021/acsapm.5c01217

**Published:** 2025-06-04

**Authors:** Daniel A. Rau, Myoeum Kim, Baoxing Xu, Li-Heng Cai

**Affiliations:** 1 Soft Biomatter Laboratory, Department of Materials Science and Engineering, 2358 University of Virginia, Charlottesville, Virginia 22904, United States; 2 Department of Mechanical and Aerospace Engineering, 2358 University of Virginia, Charlottesville, Virginia 22904, United States; 3 Department of Chemical Engineering, 2358 University of Virginia, Charlottesville, Virginia 22904, United States; 4 Department of Biomedical Engineering, 2358 University of Virginia, Charlottesville, Virginia 22904, United States; 5 Department of Chemistry, 2358 University of Virginia, Charlottesville, Virginia 22904, United States

**Keywords:** soft stretchable elastomers, vat photopolymerization printing, modular synthesis, low-cost, dissipative structures

## Abstract

Additive manufacturing of elastomers enables the fabrication of many technologically important structures and devices. However, it remains a challenge to develop soft and stretchable elastomers for vat photopolymerization (VP) printing, one of the most used additive manufacturing techniques for producing objects with relatively high resolution and smooth finishes. Here, we report a modular soft stretchable low-cost elastomer resin for VP printing. The resin consists of mainly commodity acrylates and can be photocured to form a dual-network containing covalent crosslinks and reversible double hydrogen bonds. Controlling the ratio of covalent and reversible crosslinks enables elastomers with an exceptional combination of softness and stretchability (Young’s modulus of 20–150 kPa and tensile breaking strain of 510–1350%) that cannot be achieved by existing VP resins. Using a customized VP printing platform, we transform this resin into complex three-dimensional (3D) structures. We develop an instrument to show that the 3D structures possess extreme dissipative properties, such that they can protect brain-like soft gels from impact damage in reducing the severity of impact by 75%. Together with the low cost of raw chemicals and modular nature of the design, these soft stretchable elastomer resins provide a class of feedstock for high-fidelity additive manufacturing of functional structures.

## Graphical abstract

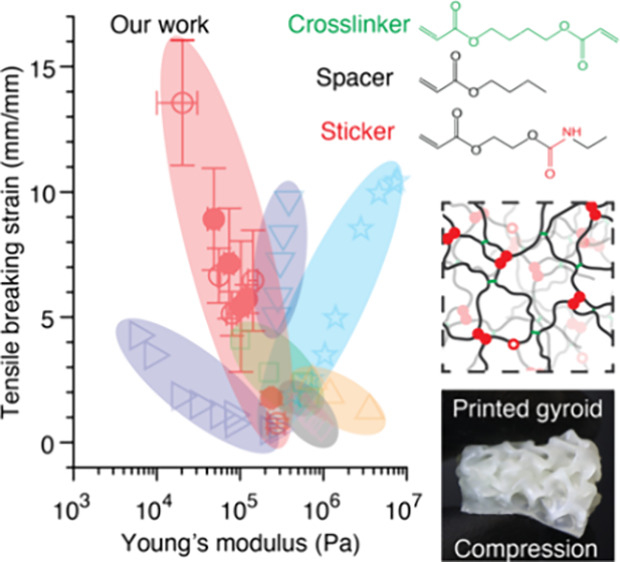

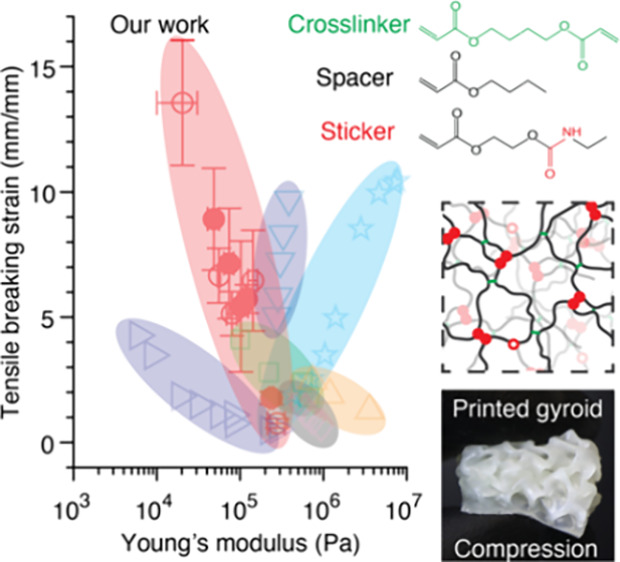

## 1 Introduction

Additive manufacturing (AM) of elastomers^1,2^ enables the fabrication of many technologically important structures and devices, such as soft robots,^3,4^ tissue scaffolds,^5,6^ stretchable electronics,^7^ sensors,^8,9^ actuators,^10–12^ and dissipative structures.^13^ Among various kinds of methods, vat photopolymerization (VP) printing represents one of the most used additive manufacturing techniques, largely because of its capability in fabricating complex three-dimensional (3D) structures with relatively high resolution and smooth finishes.^14^ However, the feedstock polymeric resins are limited by the nature of VP printing. In VP printing, a thin layer of liquid photopolymer is selectively solidified by light-activated polymerization. After the layer is cured, the build platform moves vertically, exposing the cured layer to a new layer of liquid resin. This process requires the resin to be of relatively low viscosity, such that the resin can easily flow to replenish the space between the cured part and the light source.^15^ Typically, the resin viscosity can be lowered by introducing low-viscosity diluents^16^ or solvents;^17^ however, these additives can leach out or evaporate, impairing the reliability of the printing process and/or deteriorating the mechanical properties of the printed parts. Alternatively, heating the resins reduces their viscosity during printing,^18,19^ yet the reliability of this process is limited by the poor thermal conductivity of photocurable resins. In addition, heating may promote side reactions, resulting in unpredictable crosslinking kinetics that is critical to successful printing. Through hardware engineering, a recoater is integrated into VP printers to enable printing high-viscosity resins;^20,21^ this improvement significantly broadens the selection of resins for VP printing. Nevertheless, in addition to hardware development, the advancement of VP printing requires innovation in photocurable resins.

Currently, the majority of VP resins are acrylate-based photocurable polymers, which often form highly crosslinked stiff, brittle networks.^21–23^ The commercially available photopolymers for VP printing, including variants such as Continuous Liquid Interface Production (CLIP)^24^ and Digital Light Processing (DLP),^25^ have the lowest Young’s modulus of approximately 1 MPa.^23^ The Young’s modulus of acrylate-based resins can be lowered to ∼ 500 kPa by optimizing the selection of monomers^26,27^ or to ∼ 250 kPa by using ionic liquids as fillers.^28^ However, the stiffness of these resins remains to be higher than most soft biological tissues (∼1-∼100 kPa),^29–31^ limiting their applications where contact with biological objects is required. Instead of using small acrylate-based monomers as precursors, crosslinking precursor polydimethylsiloxane (PDMS) chains by light-triggered thiol–ene click chemistry enables resins with tunable stiffness (Young’s modulus *E* of 6–283 kPa) and stretchability (tensile breaking strain ϵ_*f*_ of 50–400%).^22^ Introducing a secondary silicone-based network, which can be thermally cured post-printing, results in a dual-network with enhanced mechanical properties (*E*, 100–670 kPa; ϵ_*f*_, 180–410%).^32^ The concept of dual-network has also been extended to creating resins crosslinked by various chemistry; examples include thermally cured isocyanate network,^33^ dynamic covalent bonds,^34,35^ and reversible hydrogen bonds.^36,37^ But in general, these resins are relatively stiff and of low stretchability. Additionally, existing acrylate-based photocurable resins are relatively expensive with cost >$100 per kilogram or liter (Supporting Information, Table S1). It remains an unmet demand in the development of low-cost, soft, and stretchable elastomeric resins for VP printing.

Here, we report a modular, soft, and stretchable acrylate-based elastomer resin for VP printing. The resin can be photo-crosslinked to form a dual-network consisting of covalent crosslinks and reversible double hydrogen bonds. By controlling the ratio of covalent and reversible crosslinks, we create elastomers with unprecedented combinations of softness and stretchability (*E*, 20–150 kPa; ϵ_*f*_, 510–1350%). Moreover, the major component of the resin is commodity acrylate, which is >50 times lower in cost compared to existing ones. Using a customized VP printing platform, we transform this resin into complex 3D structures. Further, we develop an instrument to show that the 3D structures possess extreme dissipative properties, such that they can protect brain-like soft gels from impact damage in reducing the severity of impact by 75%. Together with the low cost of raw chemicals and modular nature of the design, our soft and stretchable elastomer resins provide a class of feedstock for high-fidelity additive manufacturing of functional structures.

## 2 Results and Discussion

### 2.1 Resin Design and Synthesis

We design the resin using three kinds of monomers, butyl acrylate as the “spacer”, butanediol diacrylate as the “crosslinker”, and 2-[(ethylamine)­carbonyl]­oxy]­ethyl acrylate as the “sticker” (Table S2). All three monomers are acrylate-based small molecules, which copolymerize to form an optically transparent network under ultraviolet (UV) triggered free radical polymerization in ambient air, as illustrated in Figure 1a. Both the spacer and crosslinker are commercially available; the sticker is synthesized by the reaction of isocyanate and alcohol, as detailed in **Materials and Methods** and confirmed by proton nuclear magnetic resonance spectroscopy (^1^H NMR) (Figure S1). Importantly, the major component of the resin, butyl acrylate, is a commodity with a price of ∼$1.3/kg when purchased in bulk.^38^ This is over 50 times less expensive than existing commercial elastomeric resins, which are primarily based on specialty acrylate derivatives (Table S3). Even when purchased in small quantities from standard commercial vendors (e.g., MilliporeSigma), butyl acrylate-based resins are 2–3 times less expensive than common monomers used in other photocurable resins (Table S1). Since butyl acrylate is the major component of the resin, it helps minimize the overall cost even when the more expensive sticker monomer, a small portion of the overall resin, is included. Further, the resin without stickers is 3D printable and exhibits remarkable mechanical properties. These features support that our resins are cost-competitive compared with standard commercial ones.

**Figure 1:**
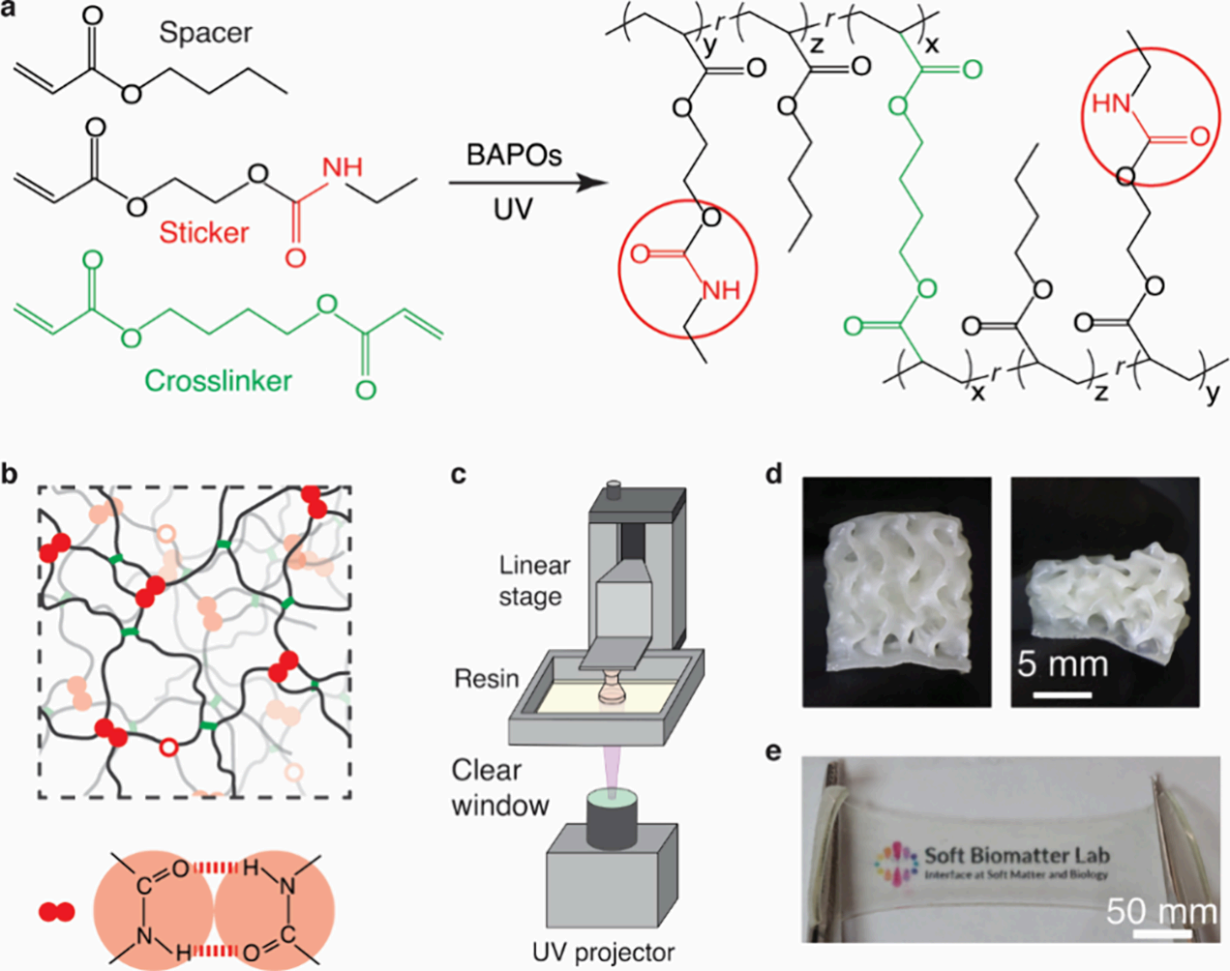
**Design and synthesis of modular soft stretchable photocurable elastomer resins for vat photopolymerization printing. (a)** The resin consists of spacer, sticker, and crosslinker monomers, which can form a network under ultraviolet (UV) triggered free radical polymerization with the help from a photoinitiator phenyl bis­(2,4,6-trimethylbenzoyl)­phosphine oxide (BAPO). (b) A schematic of the cured polymer network. Short green links: crosslinkers in (a); black lines: poly­(butyl acrylate); empty circles: amide groups; a pair of closed circles: an amide–amide double hydrogen bond. (c) Schematic of the VP printing process. Patterned UV selectively cures the resin layer-by-layer to create complex 3D objects. (d) A 3D printed gyroid structure from the elastomer resin before and during compression by hand. The part has been dyed opaque for visualization. (e) An optical image showing that the cured resin is transparent.

The sticker is essentially the same as the spacer except with one end carrying an amide group. This ensures that the sticker and the spacer have the same reaction rate, such that the sticker and the spacer are randomly distributed along the polymer chains.^39^ Moreover, a pair of stickers can form an amide–amide double-hydrogen bond,^39,40^ as illustrated by a pair of closed red circles in the lower part of Figure 1b. The randomly crosslinked network contains two types of crosslinks: (i) UV-initiated permanent, covalent bonds that crosslink two neighboring polymer chains and (ii) hydrogen bonds that act as reversible crosslinks, as shown by the upper part of Figure 1b. For this dual-network, the stiffness can be tuned by adjusting the concentration of the permanent crosslinkers and the energy dissipation efficiency can be tuned by the concentration of stickers. Thus, our design allows for modular control over the network mechanical properties.

### 2.2 Printing Complex 3D Structures

We develop a customized VP platform to print our modular soft elastomers. In our VP printer, a UV projector projects an image and selectively cures the resin to pattern each layer (Figure 1c). The selective curing process is repeated in a layer-by-layer manner to form 3D structures, as exemplified by a gyroid in Figure 1d. Unlike typical commercial VP printers that require a relatively large volume of resin, our printing platform requires a small volume of ∼1 mL, as long as the resin is sufficient to form one layer during a printing cycle. Importantly, the printing conditions can be customized to meet the crosslinking kinetics of the resin, whether the resin is optically transparent (Figure 1e) or purposely dyed to be opaque for better visualization (Figure 1d). This versatility allows for optimized curing time, critical for printing complex structures.

To determine the crosslinking kinetics of our photocurable resins, we monitor their shear moduli during exposure to UV light using a photorheometer. We adjust the irradiance of the UV to 17 mW/cm^2^, the lowest value accessible by the rheometer and is close to the 15 mW/cm^2^ irradiance used in VP printing.^41^ We use a plate–plate geometry with a gap of 100 μm, the same as the thickness of each layer during VP printing. The gelation time, *t*_*g*_, is defined as the time beyond which the storage modulus *G’* becomes larger than loss modulus *G”*, as noted by the arrow in the inset of Figure 2a. At a relatively low crosslinker concentration of 0.2% (**S0.2**), *t*_*g*_ = 30.5 s, which is too long for efficient VP printing. Slightly increasing the crosslinker concentration to 0.3% decreases the *t*_*g*_ to 23.9 s (**S0.3**). As the cross-linker concentration increases from 0.3% to 2.5%, *t*_*g*_ decreases rapidly to 4.0 s (symbols in Figure 2a, Figure S2, and Table S4).

**Figure 2:**
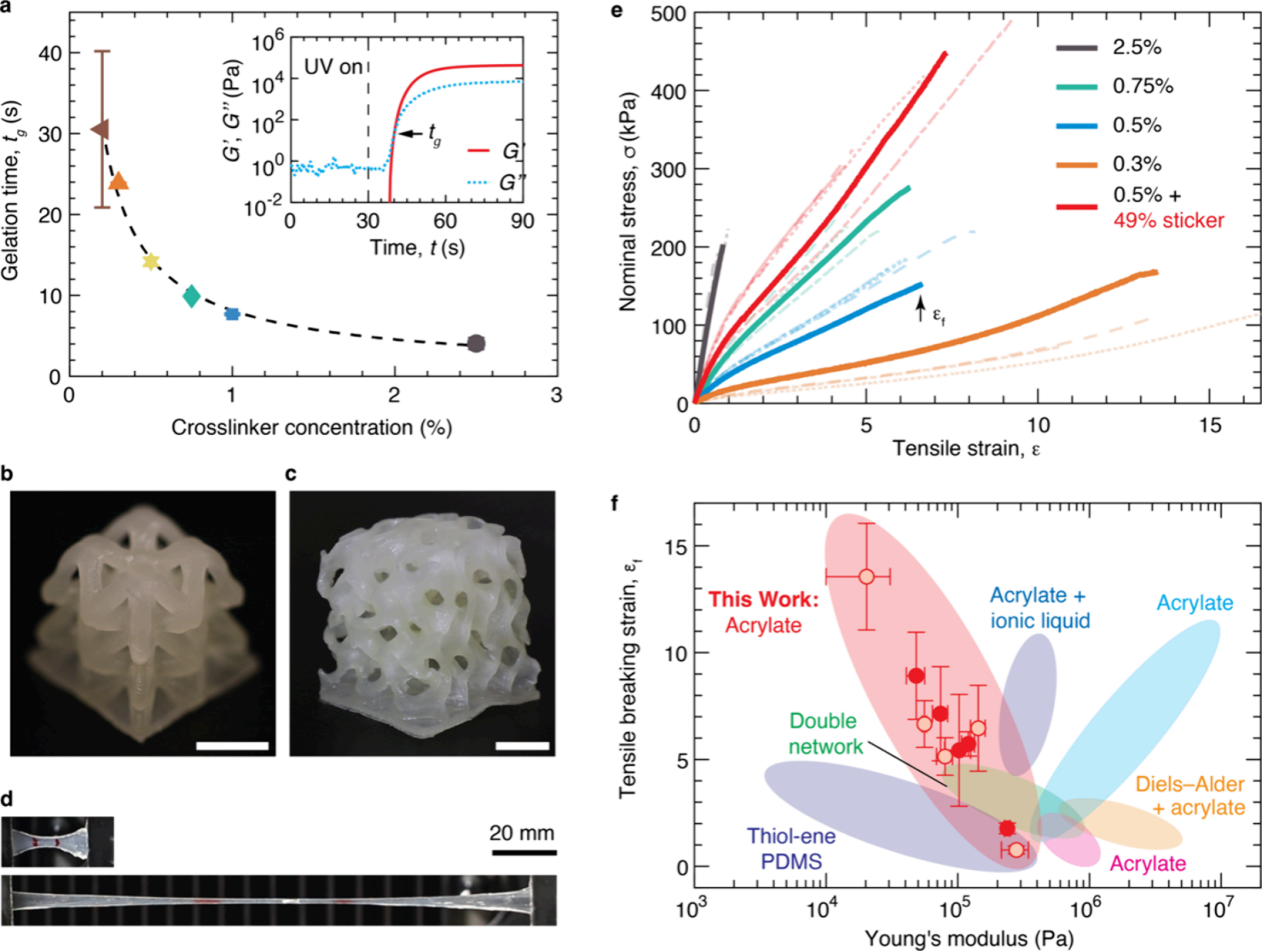
**Vat photopolymerization** **printing of soft elastomers**. (a) Dependence of gelation time after initial exposure to UV irradiation, *t*_*g*_, on the crosslinker concentration of our elastomer resins. Inset: Crosslinking kinetics for the resin formulation with 0.75% crosslinker. The gelation time is defined as the time beyond which *G’* becomes greater than *G’’*, which are quantified using small amplitude oscillatory shear measurements at a frequency of 1 Hz and a strain of 0.5% at 20 °C. The gap between two parallel plates is 100 μm, comparable to the thickness of each layer during printing. (b-c) Printed (b) 3D lattice and (c) gyroid using the resin formulation with 0.5% crosslinker. The parts are dyed opaque for visualization. Scale bars, 5 mm. (d) Photos of the printed elastomer with 0.3% crosslinker stretched to strains of 0 (upper) and 13.5 (lower). (e) Stress–strain behavior of 3D printed tensile bars using resins with various crosslinker concentrations at room temperature under a strain rate of 0.022 s^–1^. (f) Ashby plot of the Young’s modulus and tensile breaking strain of elastomer resins for VP printing. Red circles: 3D printed (empty) and molded (filled) tensile bars of our elastomer resins; light color regions: existing photocurable resins for VP printing (Table S8).

Introducing stickers decreases the gelation time compared to the resin containing only spacer monomers. For instance, for the sample with 0.5% crosslinker, replacing 49% of spacer monomers by sticker monomers reduces the gelation time by more than three times from 14.2 to 4.0 s (Figure 2a and Figure S3). This is because the stickers form reversible bonds to crosslink the polymer, effectively reducing the gelation time, as demonstrated in our previous work.^39^ These results indicate as long as crosslinker concentration is no less than 0.3%, the resins have relatively low viscosity and short curing time, allowing them to be suitable for efficient VP printing (Table S4). Indeed, these resins can be successfully printed into a lattice with truss diameter of ∼ 1 mm (Figure 2b) and a gyroid with wall thickness of ∼ 0.3 mm (Figure 2c). Moreover, the gyroid is highly deformable and can be reversibly squeezed by fingers, as shown in Figure 1d. These results demonstrate that our soft elastomer resins are suitable for VP printing of deformable, complex 3D structures.

### 2.3 Mechanical Properties

To explore the mechanical properties afforded by our elastomer resins, we conduct uniaxial tensile tests to quantify the extensibility of 3D printed tensile bars. We limit our exploration up to 2.5% crosslinker concentration, above which the resin becomes brittle and is prone to cracking upon photocuring. As the crosslinker concentration decreases from 2.5% to 0.3%, the elastomers become more stretchable with tensile breaking strain ϵ_*f*_ increasing from 76.4% to 1355.6% (Figure 2e). By contrast, the network Young’s modulus decreases linearly from 278.8 to 20.3 kPa and Figure S4. This observation is consistent with the network shear modulus, which is nearly a constant over a wide range of oscillatory shear frequency at room temperature (Figure S5) and increases linearly with the crosslinker concentration (Figure S6). Importantly, all elastomers are thermostable up to 200 °C, the highest temperature accessible by our instrument (Figure S7). These results confirm the successful development of soft, stretchable elastomer resins.

The values of Young’s moduli of printed elastomers are comparable to those of molded samples (Figure S4, Table S5 **,** and Table S8). Notably, the printed samples often exhibit a higher tensile breaking strain than the molded ones (Figure S8), which are, respectively, shown by the filled and empty circles in Figure 2f. For instance, for the elastomer with 0.3% crosslinker (**S0.3**), ϵ_*f*_ is 891.8% for the molded sample; by contrast, the printed one exhibits a remarkably high ϵ_*f*_ of 1355.6%, as visualized by the photos in Figure 2d. This difference is likely because cutting the molded samples into tensile bars introduces defects along the edges of the parts, which could lead to premature fracture of the elastomer. Additionally, we note differences in mechanical properties within samples prepared from the same resin, as shown by the light color lines in Figure 2e; this is likely because of batch-to-batch variation in resins. For instance, for the resin with 0.5% crosslinker, the difference between two batches is not significant in tensible breaking strain (665.9 ± 109.0% vs 628.9 ± 86.0%), yet becomes significant in modulus (55.8 ± 2.1 kPa vs 25.7 ± 7.8 kPa) (Figure 2e vs Figure S9, Table S7). Better understanding the cause of batch-to-batch variations and reducing variability will be the subject of future study. Nevertheless, these results show that the VP printing process does not negatively impact the mechanical properties of the printed parts.

Introducing stickers significantly enhances the mechanical properties of the printed parts. For the resin S0.5 with Young’s modulus of 55.8 kPa, replacing 49% of the spacer monomer by the sticker monomer (**S0.5–49**) increases the modulus by nearly three times to 143.0 kPa (Table S6, Figure S4). By contrast, the tensile breaking strain remains nearly constant of ∼ 650% regardless of the presence of stickers. Consequently, the tensile toughness increases by more than twice from 6.7 kJ/m^3^ to 14.5 kJ/m^3^. These results indicate that reversible associations enhance network mechanical properties.

Compared to existing acrylate-based resins for VP printing, our elastomers possess an exceptional combination of low stiffness and large extensibility (Figure 2f). For instance, solely acrylate-based resins have the minimum Young’s modulus of ∼ 400 kPa^26,27,35^ (light blue, magenta, and orange region, Figure 2f). Filling the acrylate-based resins with ionic liquids reduces the minimum modulus to 270 kPa but does not enhance stretchability (purple region, Figure 2f).^28^ Thiol–ene chemistry-based double-network resins (light green region, Figure 2f)^32^ and silicone-based resins (dark green region, Figure 2f)^22^ are softer (*E*, 6–670 kPa) but not very stretchable (ϵ_*f*_ < 430%).^22^ Commercially available resins are even more limited with typical ϵ_*f*_ < 400% and *E* > 1 MPa (Table S8). By contrast, our acrylate-based resins are both soft and stretchable (*E* < 100 kPa and ϵ_*f*_ > 500%; red circles, Figure 2f). While it is expected that the use of different printers, printing conditions, and testing conditions will result in variations in mechanical properties, our resins possess mechanical properties superior to existing ones (Figure 2f).

Because the VP printed elastomers exhibit an exceptional combination of softness and stretchability, we explore their capability to withstand large compression, which is often required for applications such as damping structures and soft robots.^42,43^ To do so, we print bulk cylinders with a diameter of 11 mm and height of 6.5 mm. We compress the cylinders at a constant strain rate of 1/30 s^–1^ and monitor the compression process using a camera. Remarkably, besides the stiffest sample (**S2.5**), which fractures at compressive strain ∼ 50%, all other elastomers can sustain up to 80% compression (Figure 3a and b). As the crosslinker concentration increases, the compressive moduli of the 3D printed elastomers increase from 88.1 kPa (**S0.3**) to 841.7 kPa (**S2.5**) (Figure 3a, Table S9). Replacing 49% of the spacer monomer with the sticker monomer in the 0.5% crosslinker material increases the compressive modulus from 209.3 kPa (**S0.5**) to 602.9 kPa (**S0.5 + 49% sticker**). In addition to being soft and deformable, the printed parts can be repeatably compressed without suffering a significant decrease in mechanical properties. For instance, when the printed gyroid (Figure 2c) is compressed to 50% strain for 100 cycles (Figure S10), the maximum stress only decreases by ∼ 5% (from 6.8 to 6.4 kPa). These results demonstrate our elastomer resins exhibit extreme mechanical properties in response to both tensile and compressive loadings.

**Figure 3:**
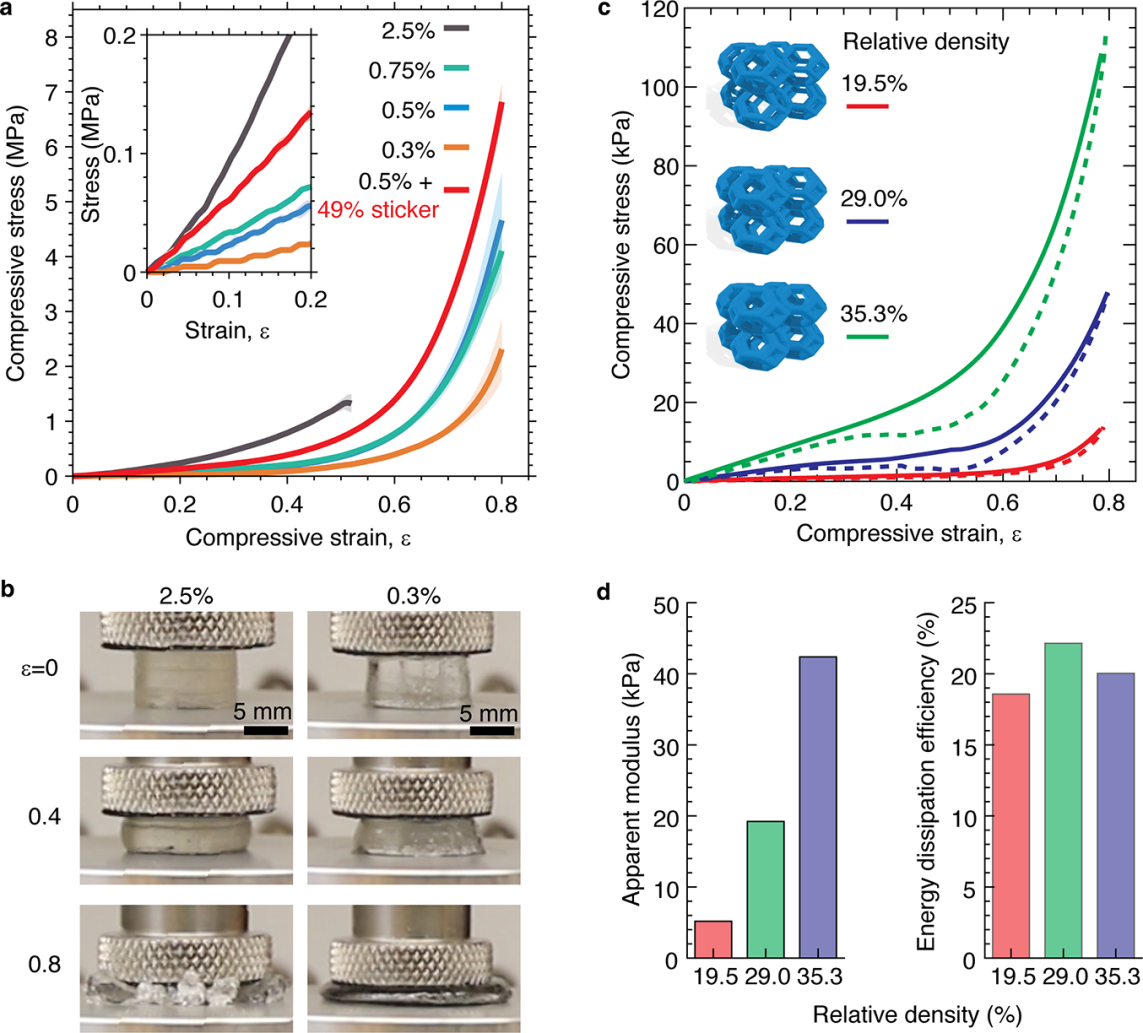
**Compression properties of printed elastomers and 3D structures.** (a) Compressive stress–strain curves of printed cylinders with various crosslinker concentrations. Inset: Zoom-in of the compressive stress–strain curves at small strains. (b) Representative photos of printed cylinders using resins with 2.5% (left) and 0.3% (right) crosslinkers. The elastomer with 2.5% crosslinker fractures at 50% compressive strain, while the elastomer with 0.3% crosslinker can be compressed to 80% strain without fracture. (c) Compressive stress–strain curves of 3D printed tetrakaidecahedron lattice structures at various relative densities. All structures are printed using the S0.5 resin. (d) Apparent modulus increases and energy dissipation efficiency remains roughly constant with increasing relative density of the 3D printed tetrakaidecahedron lattice structures.

### 2.4 Printing Dissipative Structures for Protecting Soft Brain-like Gels from Impact Damage

To highlight the potential applications of VP printing of soft stretchable elastomers, we explore the possibility in creating 3D structures with extreme dissipative structures for protecting brain-like gel from impact damage, which is critical for preventing traumatic brain injury (TBI).^44^ Depending on the location of the tissue and loading conditions, brain tissue varies in stiffness but generally has a compressive modulus between 1 and 50 kPa.^45,46^ To protect against brain injuries, a material should not only efficiently dissipate energy but also reduce acceleration during an impact. This typically requires a protective structure to be relatively soft and highly deformable (i.e., 60–80% compression strain) without dramatic stiffening.^47^ Polymer foams are commonly used to achieve this behavior^48,49^ but lack control over the 3D structure. Yet recent advancements have started to show that designing 3D structures with unique geometries promises optimum compression behavior for impacts.^50,51^

Instead of designing new 3D structures, we choose the classic tetrakaidecahedron lattices, or Kelvin foam, as protection structures; this allows for highlighting the dissipative properties enabled by the soft elastomeric resins.^52–55^ We print the **S0.5** resin into a 3D structure consisting of 2 × 2 × 2-unit cells with nominal dimensions of 2 × 2 × 2 cm. We change only the diameter of the truss members to achieve different relative densities of 19.5, 29.0, and 35.3%, which correspond to apparent compressive moduli of 5.2, 19.2, and 42.4 kPa, respectively (Figure 3c and d, Figure S11). These stiffnesses are dramatically lower than the bulk 0.5% crosslinker material (**S0.5**) with a compressive modulus of 209.3 kPa (Figure S12). Regardless of their relative density, all tetrakaidecahedron structures can be repeatedly compressed to 80% strain without fracturing (Figure 3c, Figures S13–S15). The energy dissipation efficiency, defined as the ratio of the area enclosed by the loading–unloading hysteresis loop to the area under the loading curve, is 18.6, 21.3, and 20.0% for the 19.5, 29.0, and 35.3% relative density structures, respectively (Figure 3c). Moreover, the compressive stress of the 3D structures is dramatically lower than that of the bulk counterpart. For instance, at 40% strain, the 3D structure with 29.0% relative density exhibits a stress of 6.0 kPa, nearly 25 times lower than 146.9 kPa for the bulk material (Figure S11). For the 3D printed structures, there exists a wide window of compressive strain up to ∼ 60%, within which the compressive stress is nearly plateaued. By contrast, for the bulk material, the stress starts to increase dramatically at a relatively low strain of ∼ 30%. These results highlight the ability of 3D printed soft, elastomeric architectures to be deformed by a large extent to efficiently dissipate energy without being stiffened.

To mimic the brain tissue, we use a silicone gel with a compressive modulus of 29.7 kPa (Figure S16); this gel is close to the intermediate stiffness of the brain tissue while possessing enough strength to repeatedly absorb impacts without permanent deformation. We develop an instrument to quantify in real time the ability of 3D-printed structures to protect the brain-like soft gel. In a typical measurement, we load a 3D-printed structure on top of the soft gel, which is placed on a piezoelectric force sensor, and drop a stainless-steel ball with 12.7 mm in diameter (8.35 g) from 580 mm height. Simultaneously, a high-speed camera is used to monitor the whole impact process, as illustrated in Figure 4a. The high-speed camera takes images at 826 frames per second (fps), which is sufficient for monitoring the deformation of the brain-like soft gel in real time. In parallel, the piezoelectric sensor proportionally converts the applied force to output voltage, which is recorded at a relatively high rate of 10,000/sec. A custom MATLAB program initiates and synchronizes the dropping of the ball and beginning data collection from the high-speed camera and piezoelectric sensor.

**Figure 4:**
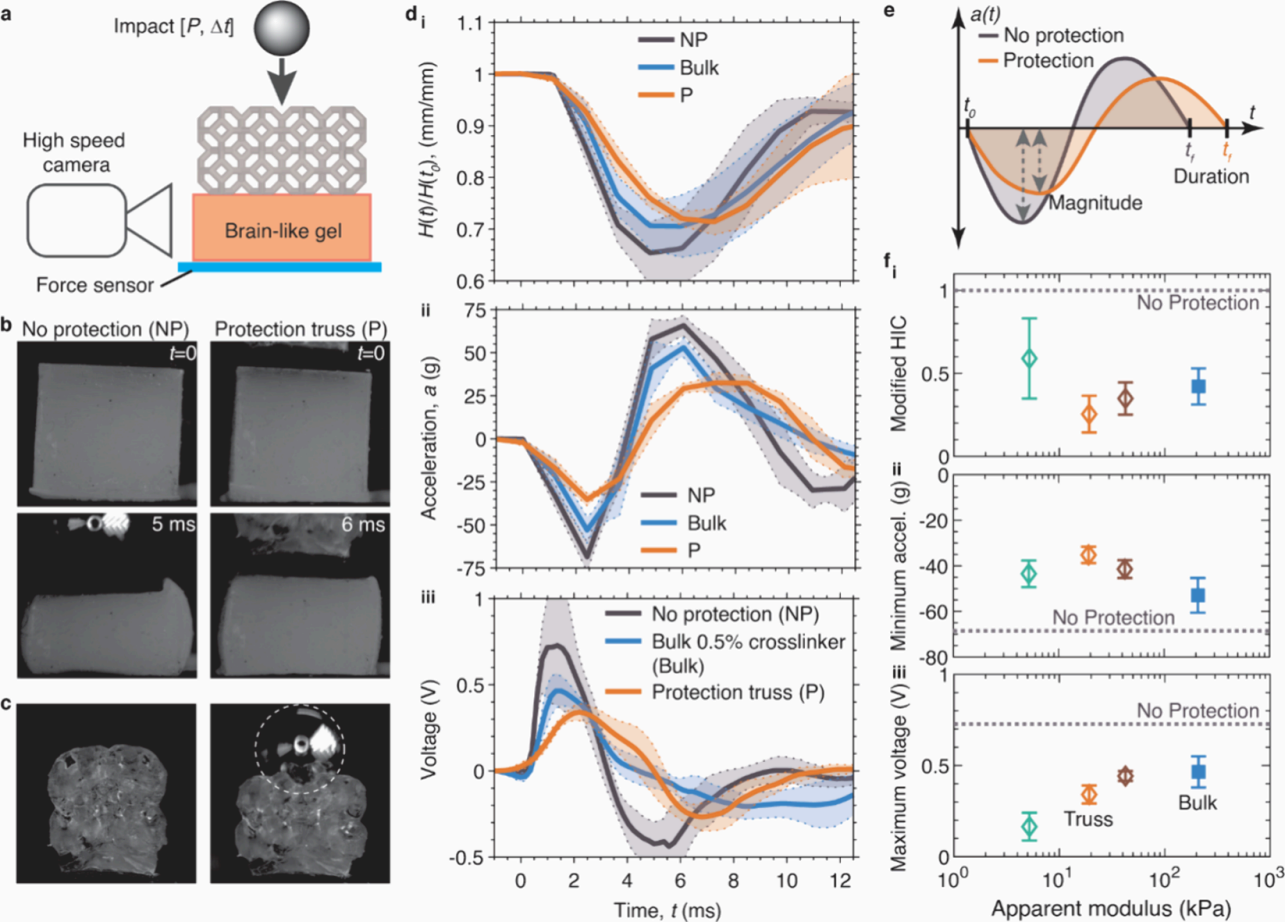
**3D printing of energy dissipative structures for protecting soft brain-like gel from impact.** (a) Experimental setup that uses a falling stainless-steel ball to produce impacts on the brain-like gel. A high-speed camera measures the deformation of the brain-like gel during impact and a piezoelectric sensor quantifies the relative magnitude of the impact force. Higher voltages correspond to higher forces. (b) Photos of the brain-like gel being deformed during impact. Left: without any protection; right: with the protection of a 3D printed tetrakaidecahedron lattice structure. (c) Photos of the 3D printed protection structure before (left) and during (right) deformation. Dashed circle outlines the falling stainless ball. (d) Strain, acceleration, and voltage (force) measured during the impact on the soft brain-like gel. Gray curves: no protection; blue curves: protected by the bulk soft, stretchable elastomer (0.5% crosslinker resin); orange curves: 3D printed tetrakaidecahedron structure with 29.0% relative density. (e) Illustration of the modified head impact criteria (mHIC) that quantifies both the magnitude and duration of the acceleration to measure the relative severity of the impact in the context of traumatic brain injury. (f) The mHIC parameter, minimum acceleration, and maximum voltage of the soft brain-like gel under the protection of the bulk soft, stretchable elastomer (0.5% crosslinker resin, squares), as well as of the printed 3D structures of different relative densities (diamonds).

During impact, both the protecting 3D structure and the soft brain-like gel rapidly deform, as shown by the high-speed images in Figure 4b,c. After impact, both the 3D structure and the gel rebound and return to their initial position. Based on the image series, we extract the height of the soft brain-like gel and calculate the average strain and acceleration associated with the gel deformation, as shown in Figure 4d and listed in Table S10. Simultaneously, we record the voltage from the piezoelectric sensor which is proportional to the impact force. The extremely short response time (∼1 ms) of the piezoelectric sensor allows for quantifying the dynamic change of force during the impact test. We repeat each measurement at least five times to ensure sufficiently powered statistics. With the protection of a 3D printed structure, the maximum extent of deformation is not only reduced but also delayed (Figure 4d,i). In delaying the impact, the protection structure reduces acceleration (Figure 4d,ii). Likewise, the voltage magnitude and thus the impact force is reduced across the whole impact tests (Figure 4d,iii). Over multiple experimental impact trials, no significant change in the impact absorption behavior or physical damage of the structures was observed. This observation indicates that the printed structures can withstand repeated deformation without notable impairment in mechanical performance (Figure S10). These results highlight the ability of 3D printed soft and stretchable elastomers to protect the soft brain-like gel from repeated impacts.

Both the magnitude and the duration of the acceleration are critical to determining the severity of a TBI.^44^ For instance, the brain can sustain high accelerations without injury but only for a very short period. Conversely, relatively low accelerations over long periods can cause severe damage. Thus, the head impact criteria (HIC)^44^ is often used to measure the likelihood of head injury arising from an impact. To quantify the difference in impact response among various protection structures, we introduce the modified head impact criteria (mHIC), a dimensionless parameter that incorporates both the magnitude and duration of the acceleration:^44^

$$mHIC=\left(t_{2}-t_{1}\right){\left[\frac{1}{t_{2}-t_{1}}\int_{t_{1}}^{t_{2}}|a\left(t\right)|dt\right]}^{2.5}$$ 1

where *t*_1_ = 0 s, *t*_2_ is the time when the ball leaves the 3D structure (∼10 ms), and *a*(*t*) is the measured acceleration over time, as illustrated in Figure 4e. The mHIC is normalized to the impact on the brain-like gel without any protection and the result, presented as a percentage, represents the relative reduction in severity of the impact. The bulk 0.5% crosslinker elastomer alone reduces the mHIC from 100% to (42.1 ± 10.9)%, the magnitude of peak deceleration decreases from 68.5 ± 8.5 g to 52.9 ± 7.6 g, and the maximum voltage decreases from 0.73 ± 0.35 V to 0.46 ± 0.09 V (Figure 4f and Table S10). Note that we use voltage to characterize the relative change of the force during impact tests because piezoelectric sensors cannot reliably measure static force (see Supporting Information, **Materials and Methods**). These results show that the inherent softness of the bulk elastomer helps reduce the severity of the impact.

Transforming the bulk soft elastomer to the even softer architected 3D structures further enhances the ability to protect the soft brain-like gel from impact (Figure S17). For instance, the magnitude of peak deceleration is reduced from 52.9 ± 7.6 g (bulk **S0.5**) to 41.4 ± 3.9 g for the structure with 35.3% relative density (Figure 4f,ii). The mHIC is reduced to (35 ± 10)%. Further decreasing the relative density to 29.0% also decreases the magnitude of peak deceleration to 35.2 ± 3.6 g and decreases the mHIC to (25 ± 11)%. For the 3D structure with the lowest relative density of 19.5% and the lowest apparent modulus of 5.2 kPa, the magnitude of peak deceleration increases to 43.5 ± 5.9 g and the mHIC increases to (59 ± 24)% (Figure 4f,i). The increase in peak deceleration is likely because the softest structure deforms too easily and does not dissipate sufficient energy during the impact, resulting in more energy being transferred to the brain-like gel than the stiffer structures. This observation highlights the importance of matching the apparent stiffness of a protective structure to the expected impact; this could be achieved by tuning the stiffness of the bulk resin and the geometry of the printed structure. Taken together, our results suggest that, in addition to materials stiffness, relative density is an important parameter in the design of the protection structures.

## 3 Conclusion and Outlook

In summary, we have developed a modular, soft, and stretchable elastomer resin for VP printing. The resin consists of three acrylate-based monomers that can be photo-crosslinked to form a dual-network, consisting of covalent crosslinks and reversible amide–amide hydrogen bonds. This design enables modular control over network mechanical properties. For instance, the Young’s modulus of the resin is linearly proportional to the concentration of crosslinkers. Introducing stickers increases network stiffness without impairing network stretchability. By controlling the ratio of covalent and reversible crosslinks, we create elastomers with an exceptional combination of softness and stretchability (*E*, 20–150 kPa; ϵ_*f*_, 500–1360%) that cannot be achieved by existing VP resins including those commercially available and reported in literature. Importantly, the major component of our resin is based on a commodity acrylate, > 50 times lower in cost compared to existing commercial ones.

Using a customized VP printing platform, we demonstrate that our resins can be transformed into complex, architecturally complex 3D structures. Because of extreme softness and stretchability of our resins, the printed 3D structures exhibit remarkable dissipative properties. The structures deform easily at relatively low stress (5–40 kPa) but without significant stiffening until a large extent of compression (60% compressive strain). We demonstrate that the soft, elastic, and dissipative structures can protect brain-like gels from impact damage by reducing the severity of impact up to 75%, highlighting potential applications in preventing traumatic brain injury.

We note that the potential of our modular resin has yet to be fully explored. For instance, reversible bonds not only slow down polymer dynamics to enhance dissipative properties,^1^ but also increase the glass transition temperature of polymers.^39^ This may allow the material to behave like stiff plastics at room temperature, providing modular resins of the same crosslinking chemistry but dramatically different mechanical properties for multimaterial VP printing.^12,56^ The only sticker concentration explored in this work is 49%. Systematic investigations into the roles of hydrogen bonds on the mechanical properties of the resins and the dissipative properties of the printed 3D structures will be the subject of future explorations. Additionally, incorporating ionic liquids may allow the resins to be electrically conductive, resulting in soft, stretchable, and conductive elastomers.^28,57,58^ Although VP printed structures made of our soft and stretchable resins can sustain repeated large compression (Figure S10), it would be interesting to explore the long-term performance and durability of these materials and structures under cyclic high strain loading.^59^ Note that the volatility of butyl acrylate may affect the printing reliability. To mitigate this, in our experiments, we synthesize fresh batches of resin before printing. It would be interesting to explore the effects of resin volatility on the reliability of printing as well as to identify ways to reduce the volatility of the resin. Butyl acrylate often has an unpleasant odor; thus, our manufacturing process is performed inside a fume hood. Yet, it is possible to use alternative butyl acrylate monomers to minimize the odor while retaining mechanical properties.^60^ Together with the low cost of raw chemicals and extreme mechanical properties, our modular elastomer resins provide a class of soft and stretchable materials for high-fidelity additive manufacturing of functional structures and devices.

## Supplementary Material



## Data Availability

All data are available in the manuscript or the Supporting Information.

## Notes

Detailed experimental procedures include materials, monomer synthesis, resin preparation, 3D printing, mechanical testing, and impact tests. Figures S1–S17 further detail material characterization, mechanical properties, and impact performance. Tables S1–S10 detail material costs, formulations, curing behavior, mechanical properties, batch consistency, literature comparisons, and impact performance. (PDF)
