# Supplementary material for: Vat Photopolymerization Printing of Modular Soft Stretchable Low-Cost Elastomers
Source: ACS Appl Polym Mater. 2025 Jun 4;7(11):7566–74. doi: 10.1021/acsapm.5c01217 (PMC12172013; doi:10.1021/acsapm.5c01217)
Supplement: Supplementary file 1 [file ap5c01217_si_001.pdf]

Supporting Information

Vat photopolymerization printing of modular soft stretchable low-cost elastomers

Daniel A. Rau[1, +,†], Myoeum Kim[1,†], Baoxing Xu[2], Li-Heng Cai[1,3,4,5,*]

Affiliations:

[1]Soft Biomatter Laboratory, Department of Materials Science and Engineering, University of Virginia, Charlottesville, VA 22904, USA

[2]Department of Mechanical and Aerospace Engineering, University of Virginia, Charlottesville, VA 22904, USA

[3]Department of Chemical Engineering, University of Virginia, Charlottesville, VA 22904, USA

[4]Department of Biomedical Engineering, University of Virginia, Charlottesville, VA 22904, USA

[5]Department of Chemistry, University of Virginia, Charlottesville, VA 22904, USA

† These authors contribute equally

+ Present address: Department of Mechanical Engineering, University of Wyoming, Laramie, WY 82072, USA

*Corresponding author. Email: liheng.cai@virginia.edu

Corresponding author contact:

Dr. Li-Heng Cai
228 Wilsdorf Hall
University of Virginia
395 McCormick Road
Charlottesville, VA 22904
Tel: 434-924-2512
Fax: 434-982-5660

Table of Contents

Vat photopolymerization printing of modular soft stretchable low-cost elastomers 1

Materials and Methods 4

Figure S1. H NMR spectrum (600 MHz, $CDCl_3$) of the synthesized 2-[[ethyl(amine)carbonyl]oxy]ethyl acrylate (EAEA) sticky monomer. 8

Figure S2. Crosslinking kinetics for resins with different crosslinker concentrations. 9

Figure S3. Photorheology of the resin with 0.5% crosslinker and 49% stickers. 10

Figure S4. Linear relationship between Young's modulus (tensile) and crosslinker concentration for 3D-printed samples. 11

Figure S5. Frequency sweep of elastomers with different crosslinker concentrations. 12

Figure S6. The shear storage modulus of the resins increases linearly with crosslinker concentration. 13

Figure S7. Temperature sweep of elastomers with different crosslinker concentrations. 14

Figure S8. Tensile properties of molded elastomers. 15

Figure S9. Tensile properties of two separate batches of the 0.5% crosslinker samples. The two batches were synthesized, printed, and tested separately. 16

Figure S10. Repeated cyclical compression to 50% strain of a gyroid printed from the resin containing 0.5% crosslinker. 17

Figure S11. Cyclic compression test of 3D printed tetrakaidecahedron structures. 18

Figure S12. Comparison of the bulk elastomer to 3D structures with various relative density. .. 19

Figure S13. Cyclic compression test for the impact absorption structure with 19.5% relative density up to compressive strain of 80%. 20

Figure S14. Cyclic compression test for the impact absorption structure with 29.0% relative density up to compressive strain of 80%. 21

Figure S15. Cyclic compression test for the impact absorption structure with 35.3% relative density up to compressive strain of 80%. 22

Figure S16. Cyclic compression of brain tissue mimicking soft gel. 23

Figure S17. Strain, acceleration, and voltage (force) measured during the impact on the tetrakaidecahedron protection structures. 24

Table S1. Price of commonly used monomers in photocurable resins 25

Table S2. Formulations of soft, stretchable elastomeric resins for VP printing. 25

Table S3. Cost of commercially available elastomeric resins for VP printing. 26

Table S4. UV curing properties. 27

Table S5. Tensile properties of molded elastomers. 27
Table S6. Tensile properties 3D printed elastomers. 27
Table S7. Batch-to-batch comparison of 0.5% crosslinker samples. 27
Table S8. List of data points in Figure 2F. 28
Table S9. Compression properties of our 3D printed resins. 29
Table S10. Characteristics of 3D printed structures under impact. 29
References 30

Materials and Methods

Materials. Ethyl isocyanate (98%), 2-hydroxyethyl acrylate (96%), hydroquinone (≥99%), dibutyltin dilaurate (DBTDL, 95%), butanediol diacrylate (BDDA, 95%), and phenylbis(2,4,6-trimethylbenzoyl)phosphine oxide (BAPOs, 97%) were purchased from Sigma Aldrich. Butyl acrylate (BA, >99%) and avobenzone (>98%) were purchased from TCI. All chemicals are used as received unless notified otherwise.

Synthesis of sticky monomer 2-[[ethyl(amine)carbonyl]oxy]ethyl acrylate (EAEA). The sticky monomer is synthesized using alcoholysis reaction. First, a round flask is settled with a condenser for reflux. The flask is charged with ethyl isocyanate (25g, 0.35 mol), DBTDL (125 mg, 0.05% mass fraction of isocyanate), and hydroquinone (77 mg, 0.2% mole fraction of isocyanate) under nitrogen. The flask is sealed and stirred for 15 min in an ice bath. The ice bath is replaced by an oil bath and then 2-hydroxyethyl acrylate (40.642 g, 0.35 mol) is added dropwise with vigorous stirring under nitrogen at 40 ^{o}C. After finishing the addition of 2-hydroxyethyl acrylate, the reaction mixture is stirred at 60 ^{o}C for 4 hours. The success of the synthesis is confirmed by ^{1}H NMR (600 MHz, $CDCl_3$) δ=1.00 (t, 3H), 3.17 (m, 2H), 4.25 (m, 4H), 4.87 (s, 1H), 5.82 (d, 1H), 6.10 (dd, 1H), 6.38 (d, 1H) (**Figure S1**).

Preparation of photocurable resin. The soft photocurable resin is prepared by mixing BA, BDDA, and BAPOs at the ratios described in **Table S1**. Avobenzone was added into the mixture at 0.1 wt.% of total resin as a UV absorber to improve the printing quality by reducing the scattering of light. Afterward, the mixture was bubbled with nitrogen for 30 min to remove oxygen under vigorous stirring. The resin containing sticky monomers is prepared by mixing BA, EAEA, BDDA, and BAPOs at the ratios listed in **Table S1**.Again, avobenzone was added at 0.1wt.% of total resin and the mixture bubbled with nitrogen for 30 min to remove oxygen under vigorous stirring.

Vat photopolymerization 3D printing. Vat photopolymerization (VP) 3D printing was completed on a custom built system consisting of a Wintech PRO6500 UV Projector (385 nm), a Zaber X-LSQ150A linear stage, and a Sovol resin vat with a clear fluorinated ethylene propylene

(FEP) window. Printing was completed using 100 μm layer thickness and a UV irradiance set to 17 mW/cm^2. Layer UV Exposure times for each resin were determined through experimention with a 5 sec expsoure time per layer being used for the 2.5% crosslinker sample, 9 sec for the 0.75% crosslinker, 12 sec for the 0.5% crosslinker, and 15 sec for the 0.3% crosslinker. Variation in the gelation time measured with photorheology and the exposure times used in printing is primarily due to the slight difference in the UV sources between the printer and the photorheoemter. The photorheometer is equipped with a broad range UV source with an installed filter that reduces the wavelength range to between 320 and 395 nm. Conversely, the UV projector has a 385 nm LED UV source. After printing, parts were washed with isopropanol alcohol and further cured in a Formlabs Form Cure system at 60 ºC for one hour.

Dynamic mechanical tests. Tensile and compression testswere completed on a Mark-10 ESM303 motorized test stand. A 10 N load cell was used for tensile testing and a 50 N load cell was used for compression testing. The test stand was set to the lowest speed possible, 13 mm/minute, corresponding to an average strain rate of 0.052 s^{-1} for the 3D-printed samples and 0.022 s^{-1} for the cast samples.

Photorheology, frequency sweeps, and temperature sweeps were completed on astress controlled rheometer (Anton Paar MCR302) to measure the curing behavior of the resins and mechanical properties of the cured materials. For photorheology, an 8mm diameter parallel plate geometry, a 100 μm gap, 17 mW/cm^2 UV irradiance, 0.5% strain, 1 Hz frequency, and the photocuring accessory were used. UV exposure began 60s into the experiment to allow the resin time to equilibrate and a total of 300s of UV exposure was used. For the frequency sweeps, an 8mm diameter parallel plate geometry, a 100 μm gap, 0.5% strain, 20 °C constant temperature, and a frequency range from 0.1 to 100 Hz were used. For the temperature sweep, an 8mm diameter parallel plate geometry, a 100 μm gap, 0.5% strain, and 1 Hz frequency were used. The temperature was ramped from -40 to 200 ºC at a rate of 3 ºC/min.

Impact Studies. Impact studies were completed using the experimental setup illustrated in Figure 4a. A Fastec IL5-S high speed camera was used to record images at 826 fps. A long working distance objective was focused on the brain tissue mimicking soft gel. The light-colored silicones

contrasted with the much darker surroundings. An image processing program was developed in Matlab to extract the height of the soft gel over time by thresholding the brightness of each pixel in the image. Pixels with a grayscale brightness of over 40 out of 255 were used to identify the dimensions of the soft gel. The height of the soft gel was averaged over the center 50% of the image to account for any local variation or noise.

A 10×17 mm poly(vinylidene fluoride) (PVDF) piezoelectric sensor from MEAS was used to measure the force of the impacts. The voltage output from the sensor was read directly using a National Instruments USB-6003 data aqcuisition device recoridng at 10 kHz. A 1 MΩ resistor was used to protect the device from over voltage damage. Typically, a piezoelectric sensor begins to discharge its voltage rapidly after being strained and is not capable of providing steady state measurements. However, the short time scales of the impacts (less than 15 ms) makes the piezoelectric sensor suitable for measuring the force of these impacts. Therefore, we decided to use voltage to characterize the relative change of the force during impact tests. The piezoelectric sensor was fixed to a stiff table. The soft gel and protection stuctures were then attached using silicone grease. This grease provides the necessary adhesion while allowing the soft gel to freely deform under impact.

To synchronize various data sources, we used a data acquisition device (DAQ) to continously monitor the state of the electromagnet. When the DAQ sensed that the electromagnet was turned off, meaning that the steel ball began dropping, the DAQ sent a signal to the camera to begin recording and at the same time began recording of the voltage signal. Simulatenosuly, the high-speed camera is used to capture the deformation of the soft gels.

The silicone based brain-mimicking soft gels was created by adding Smooth-On Slacker at 20 wt% to Smooth-On Ecoflex 00-20 and mixing vigorously. The material was then cured at 80 °C for 24 hours.

When calculating the modified Head Impact Criteria (mHIC) (eq. 1, main text), the magnitude and duration of the acceleration between the time the falling ball first contacts the sample and the time when the sample rebounds to its initial position was considered. Analysis of the impact images

shows that when the sample returns to its initial height the ball stops contacting the sample and is ejected. At the same time, the acceleration is equal to zero. This occurs between approximately 10 and 12 ms in experiments.

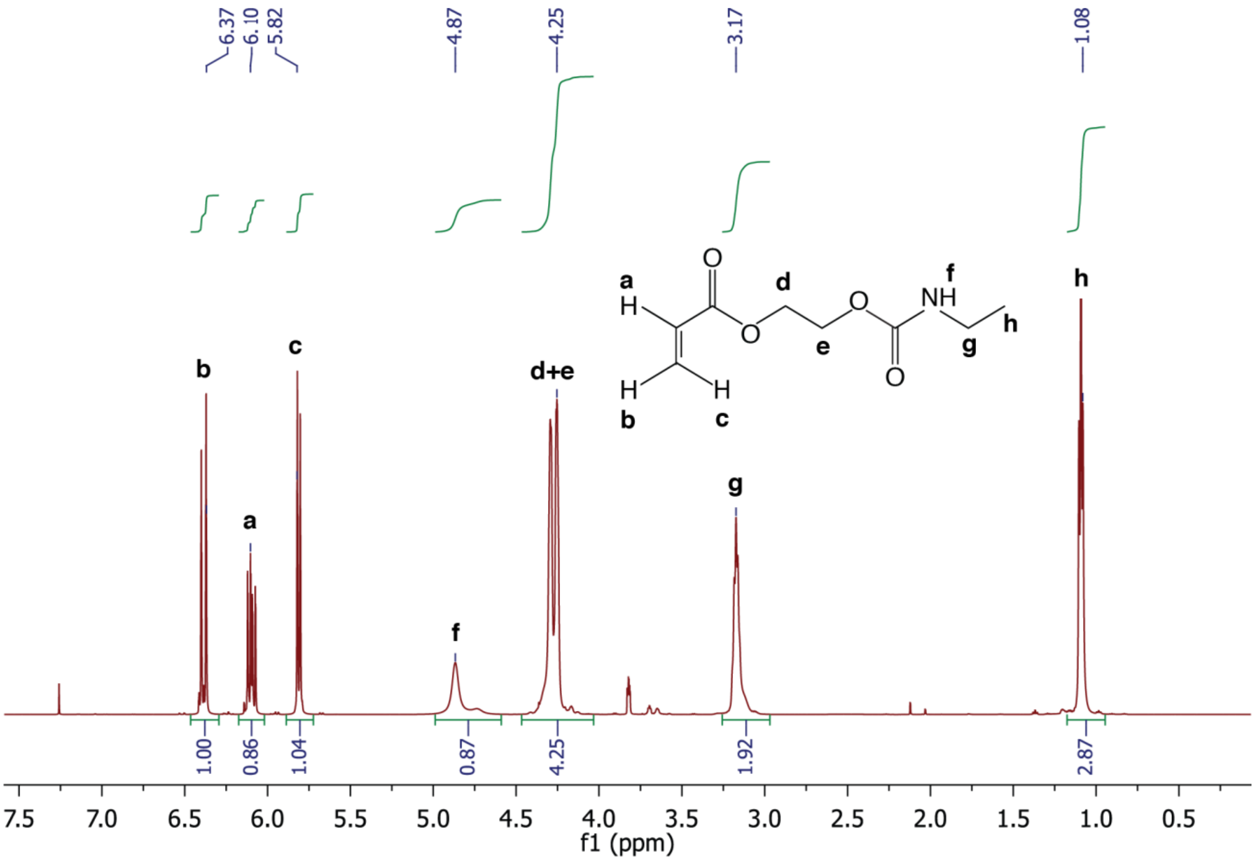

Figure S1. H NMR spectrum (600 MHz, $CDCl_3$) of the synthesized 2-[[ethyl(amine)carbonyl]oxy]ethyl acrylate (EAEA) sticky monomer.

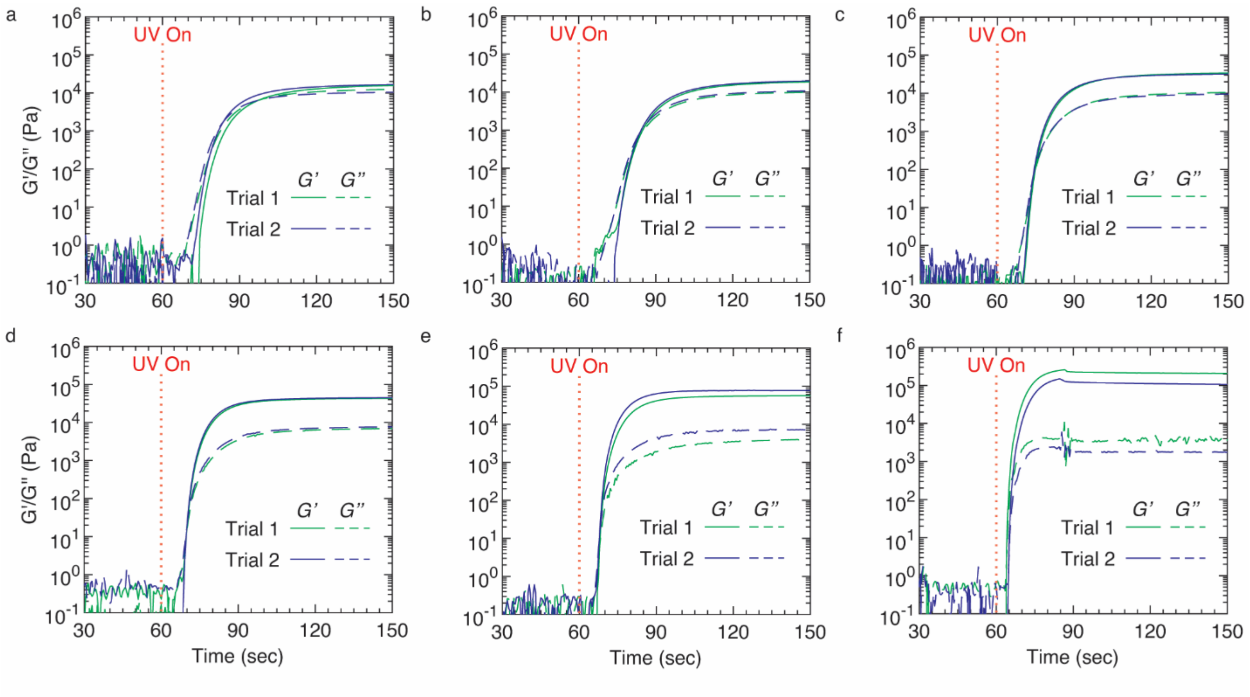

Figure S2. Crosslinking kinetics for resins with different crosslinker concentrations. Higher crosslinker concentration results in shorter gel time and higher modulus. (a) 0.2% crosslinker (b) 0.3% crosslinker (c) 0.5% crosslinker (d) 0.75% crosslinker (e) 1.0% crosslinker (f) 2.5% crosslinker. A 100 μm gap and 17 mW/cm^2 irradiance was used for all measurements.

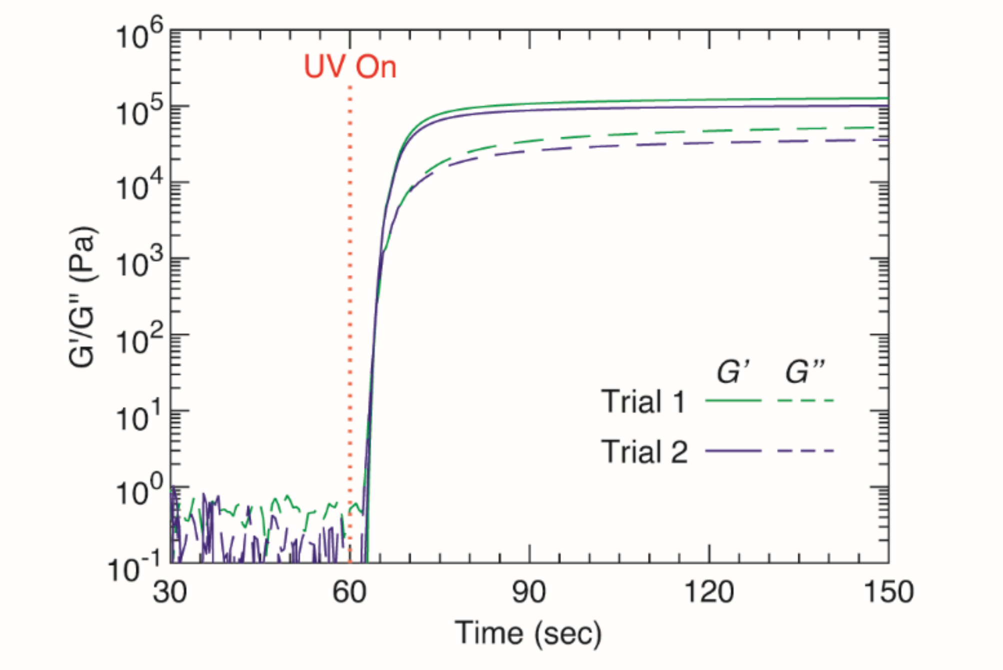

Figure S3. Photorheology of the resin with 0.5% crosslinker and 49% stickers.
Compared to the resin containing only 0.5% crosslinker (100% spacer monomer), the resin with sticker has a shorter gelation time and a higher modulus.

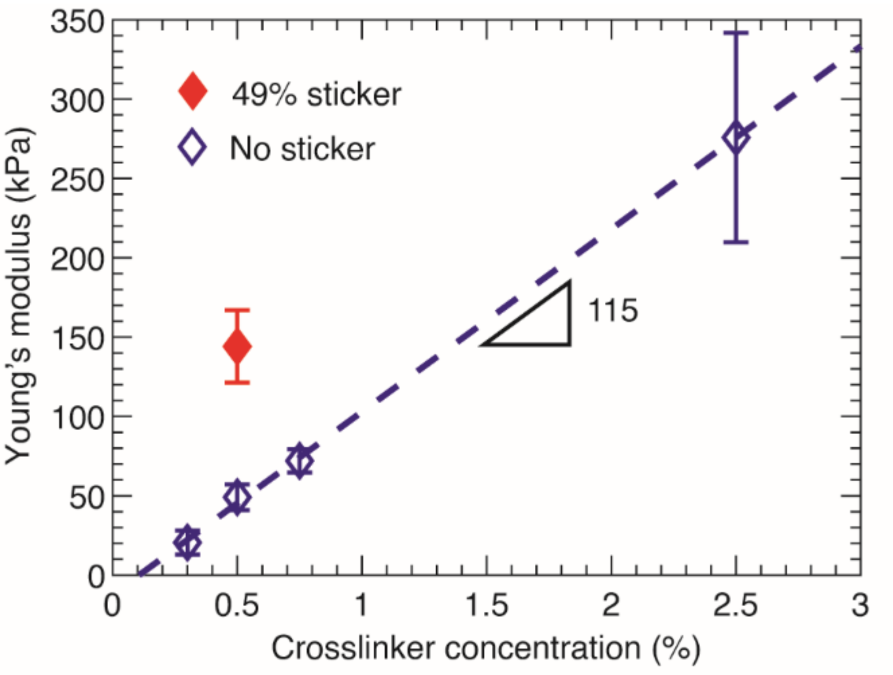

Figure S4. Linear relationship between Young's modulus (tensile) and crosslinker concentration for 3D-printed samples.

Filled red symbol represents the sample containing 49% sticker monomer. All measurements are performed at a strain rate of 0.052 s^{-1}.

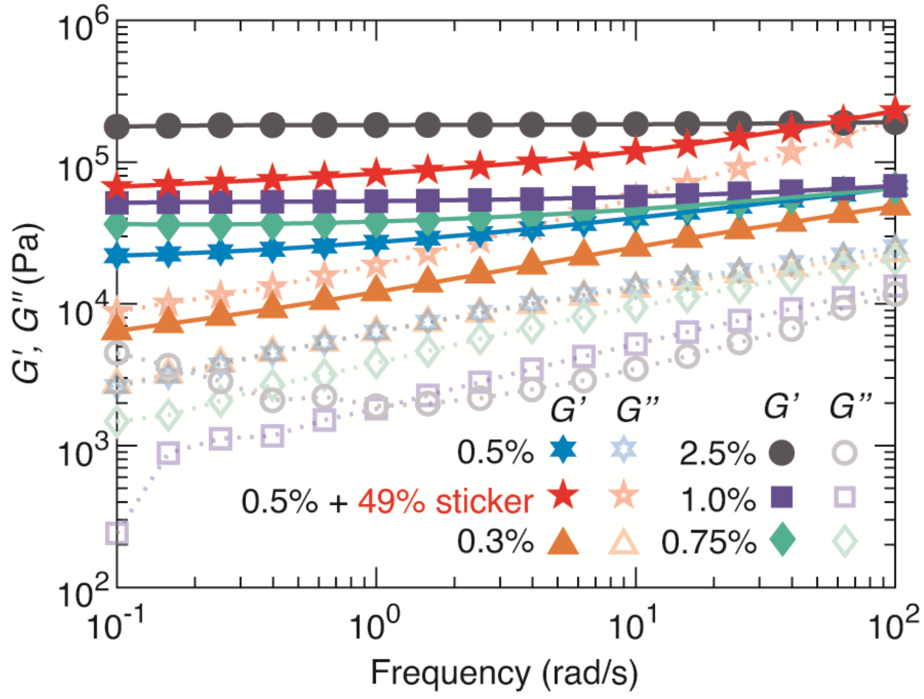

Figure S5. Frequency sweep of elastomers with different crosslinker concentrations. Dependencies of storage (solid symbols, *G'*) and loss (open symbols, *G''*) moduli on the oscillatory shear frequency. All measurements are performed a fixed strain of 0.5% at 20°C.

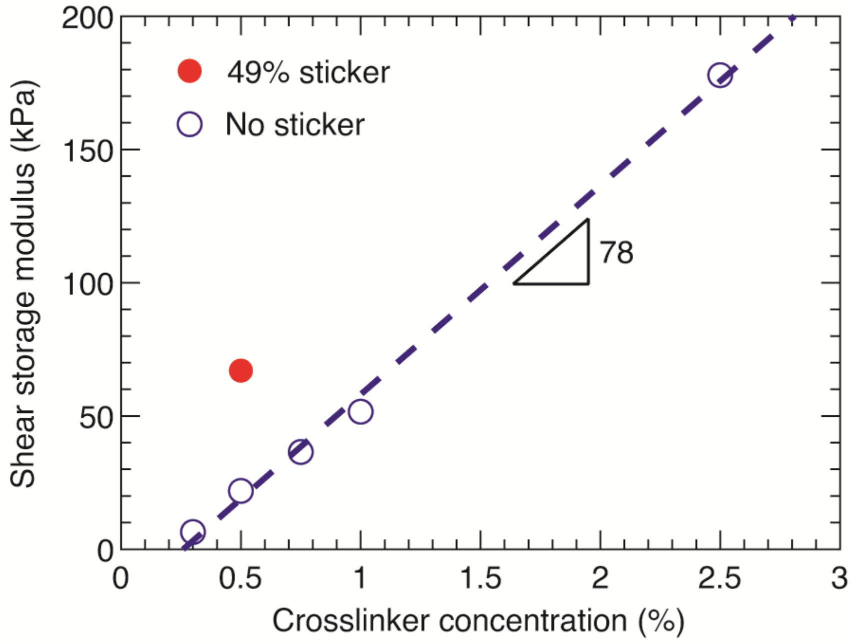

Figure S6. The shear storage modulus of the resins increases linearly with crosslinker concentration.

Filled red symbol represents the sample containing 49% sticker monomer. All measurements are performed at 0.5% strain and oscillatory frequency of 0.1 rad/s.

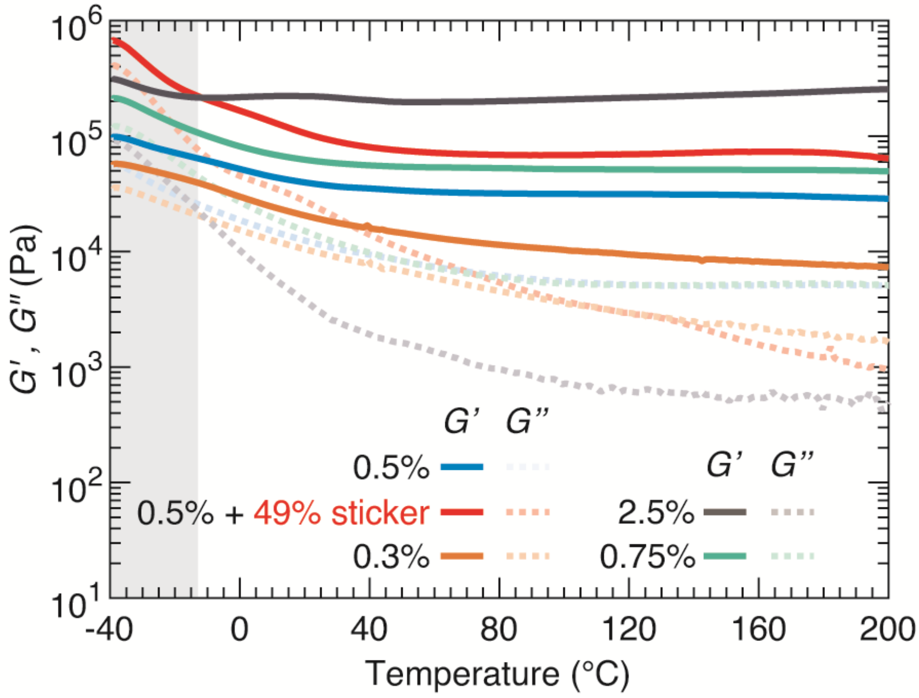

Figure S7. Temperature sweep of elastomers with different crosslinker concentrations. Temperature dependence of *G'* (solid line) and *G''* (dashed line) from -40 °C to 200 °C. All measurements are performed a fixed strain of 0.5% and 1 Hz.

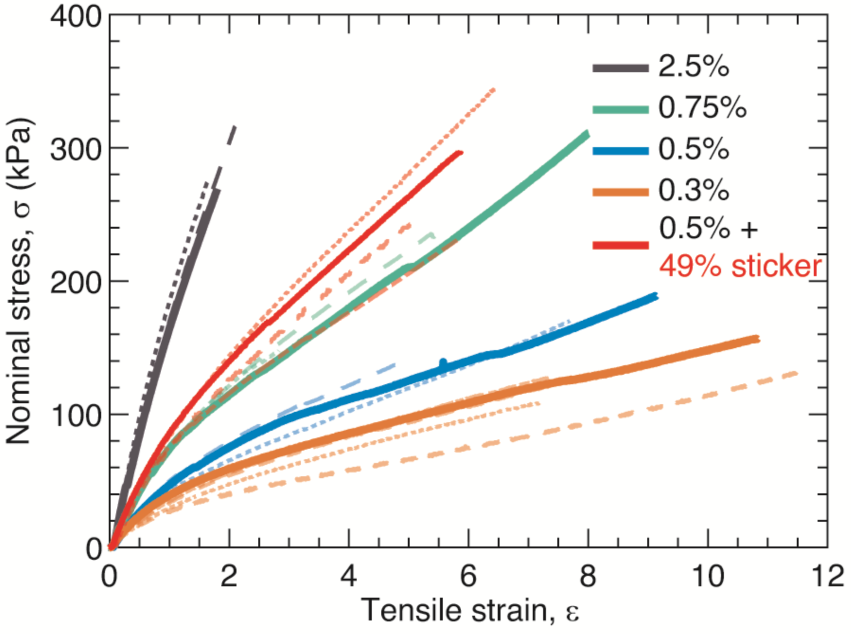

Figure S8. Tensile properties of molded elastomers.

Stress-strain behavior of cast tensile bars using resins with various crosslinker concentrations at room temperature under a strain rate of 0.022 s^{-1}.

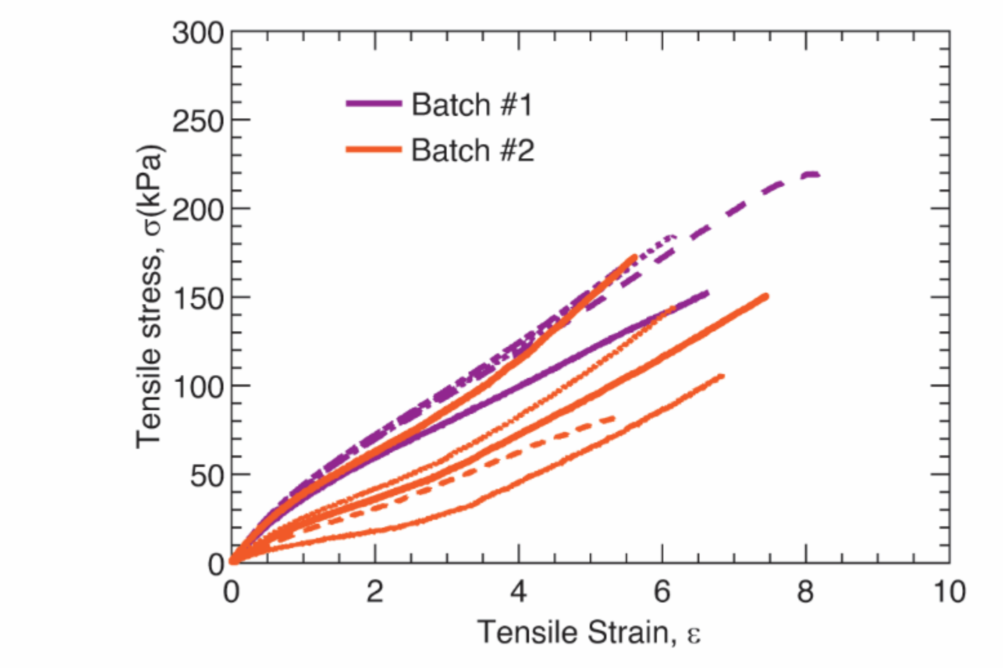

Figure S9. Tensile properties of two separate batches of the 0.5% crosslinker samples. The two batches were synthesized, printed, and tested separately.

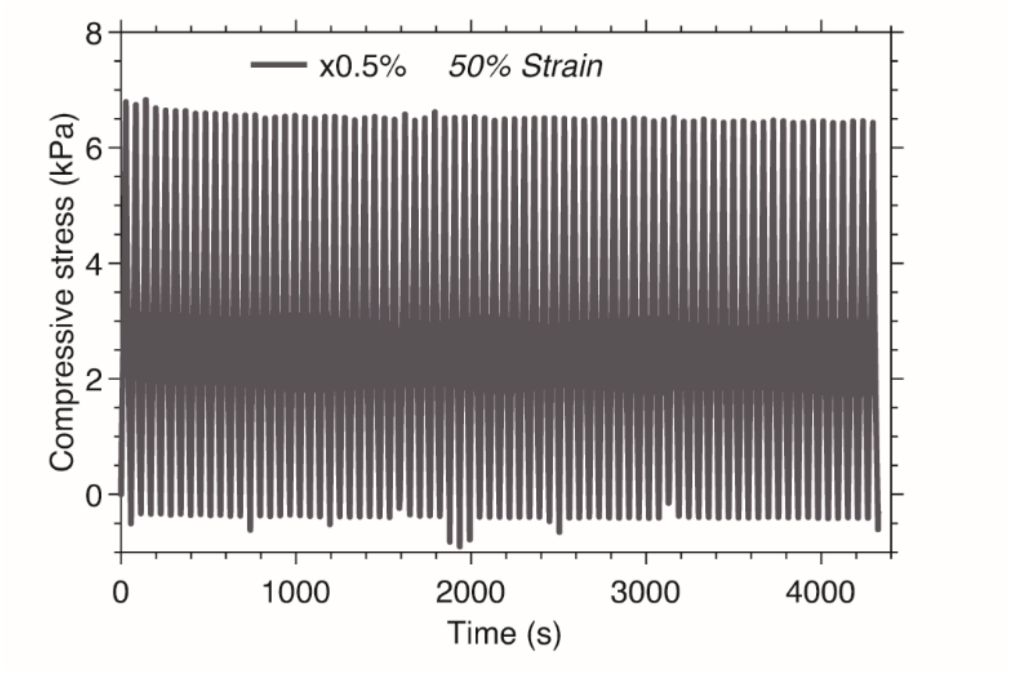

Figure S10. Repeated cyclical compression to 50% strain of a gyroid printed from the resin containing 0.5% crosslinker.

The printed part can be repeatably deformed without suffering a significant decrease in mechanical properties.

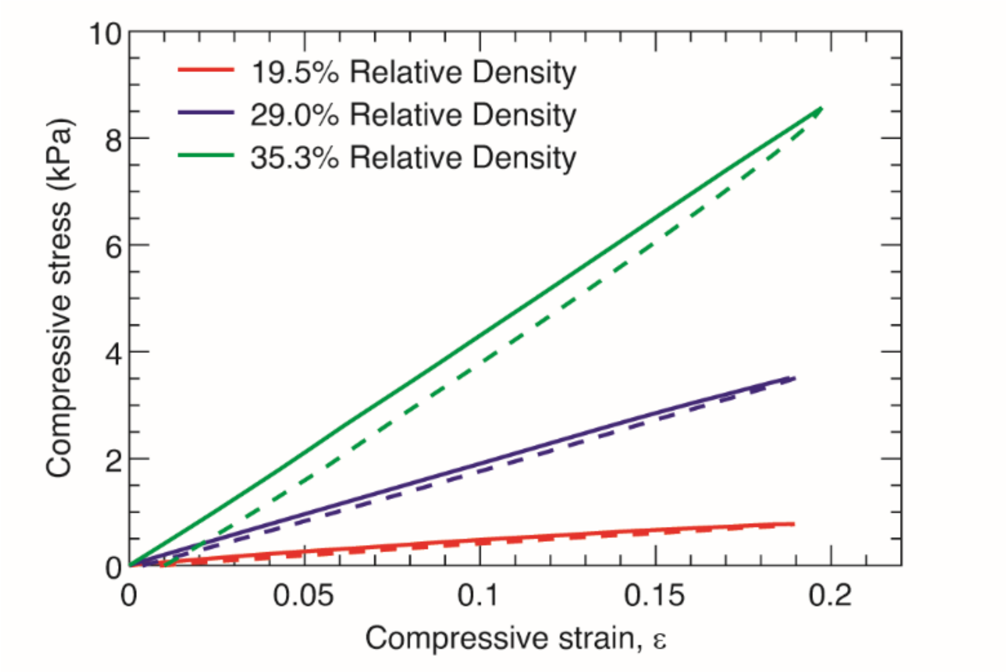

Figure S11. Cyclic compression test of 3D printed tetrakaidecahedron structures.

The 19.5% relative density structure has a compressive modulus of 5.2 kPa, the 29.0% relative density structure has a modulus of 19.2 kPa and the 35.3% relative density structure has a modulus of 42.4 kPa recorded at 0.05 strain. All structures are printed from the resin containing 0.5% crosslinker (S0.5) and are used to protect soft materials from impacts. Solid line: loading; dashed line: unloading.

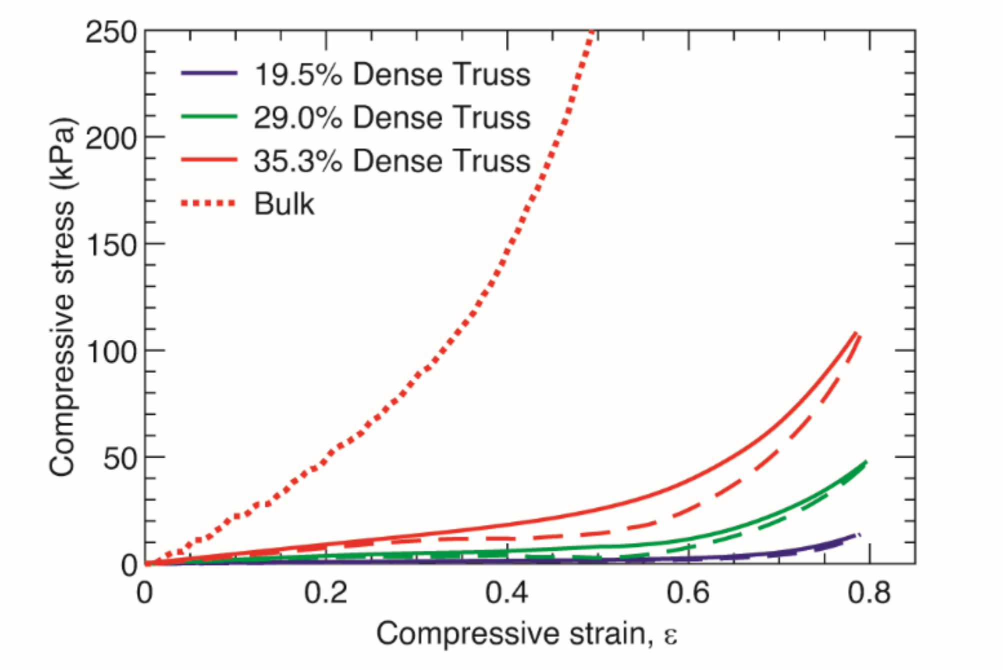

Figure S12. Comparison of the bulk elastomer to 3D structures with various relative density. The 3D printed impact absorption structures are softer than the bulk material and can be strained to 80% deformation with minimal plastic deformation. All structures are printed using the **S0.5** resin.

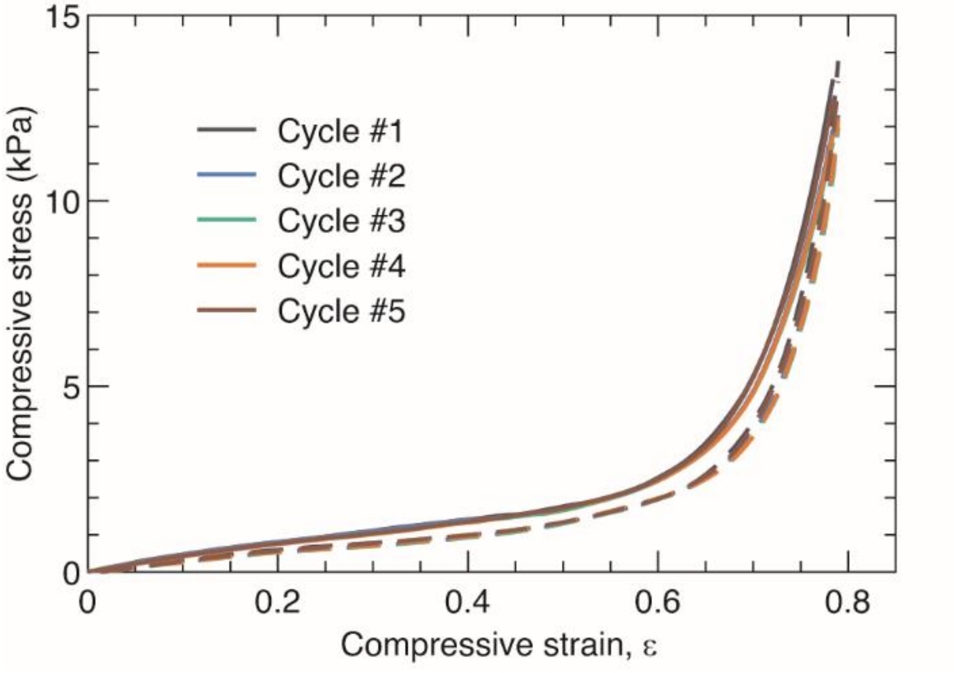

Figure S13. Cyclic compression test for the impact absorption structure with 19.5% relative density up to compressive strain of 80%.

The structure printed from the resin containing 0.5% crosslinker can repeatably deform to 80% strain without experiencing permanent deformation or breaking. The structure exhibits an energy dissipation efficiency of 18.6%.

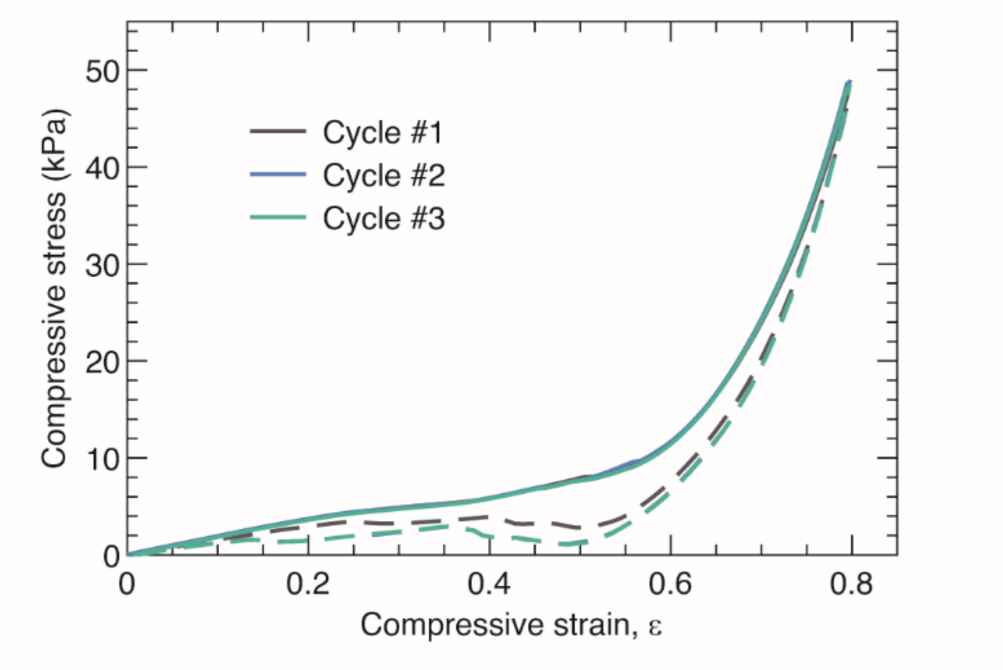

Figure S14. Cyclic compression test for the impact absorption structure with 29.0% relative density up to compressive strain of 80%.

The structure printed from the resin containing 0.5% crosslinker can repeatably deform to 0.8 strain without experiencing permanent deformation or breaking. The structure exhibits an energy dissipation efficiency of 21.3%.

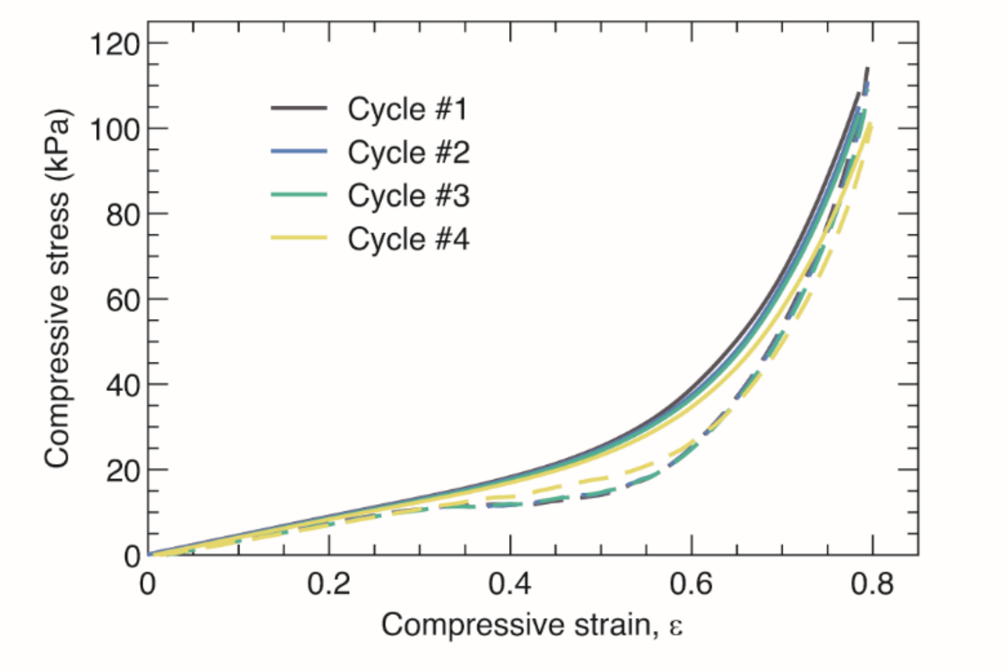

Figure S15. Cyclic compression test for the impact absorption structure with 35.3% relative density up to compressive strain of 80%.

The structure printed from the resin containing 0.5% crosslinker can repeatably deform to 0.8 strain without permanent deformation or breaking. The structure exhibits an energy dissipation efficiency of 20.3%.

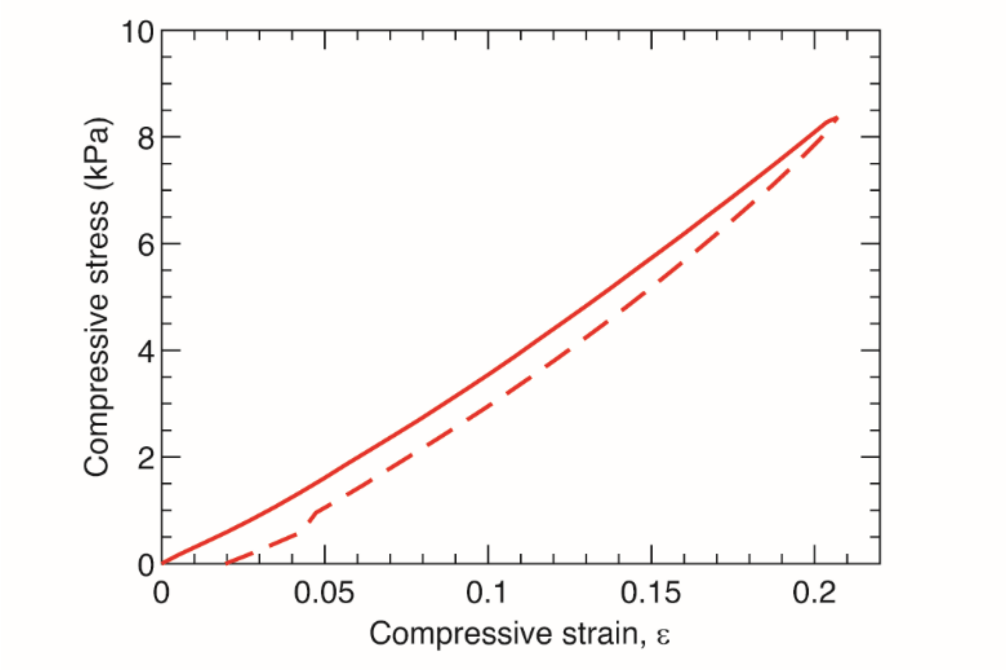

Figure S16. Cyclic compression of brain tissue mimicking soft gel.

Soft silicone gel has a compressive modulus of 29.7 kPa measured at 0.02 strain. Solid line: loading; dashed line: unloading.

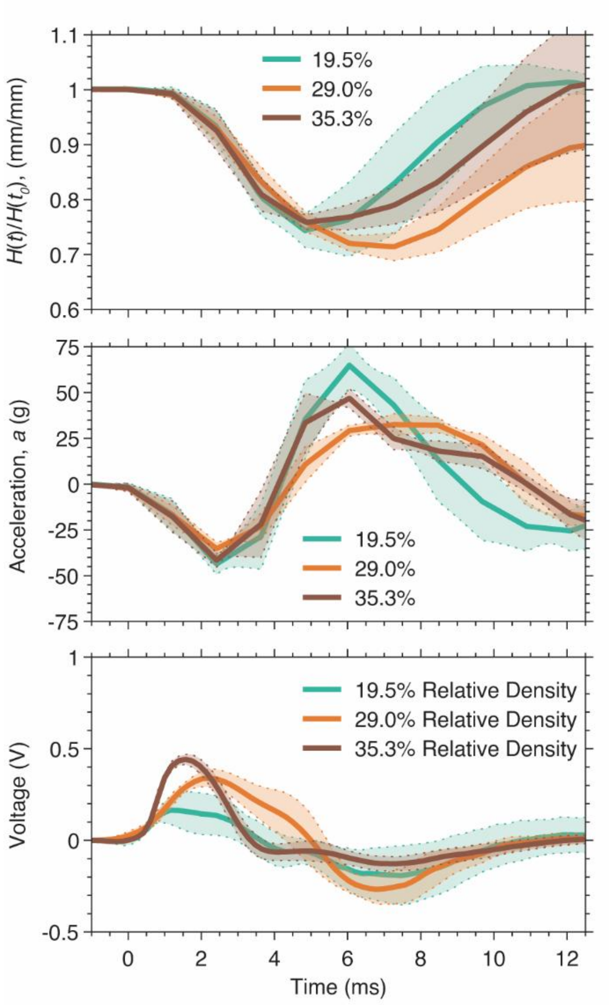

Figure S17. Strain, acceleration, and voltage (force) measured during the impact on the tetrakaidecahedron protection structures.

The 3D structures printed from the resin containing 0.5% crosslinker (S0.5) reduces the strain, acceleration, and voltage (force) of the impact. The absorption structures protect the brain-like material by helping to dissipate the impact forces, a unique property enabled by both the softness of the bulk material and the geometry of the 3D printed structures. The impact response varies with the design and the resulting stiffness of the trusses.

Table S1. Price of commonly used monomers in photocurable resins

List of commonly used monomers for photocurable resins provided by Mendes-Felipe et al. (2019)[1]. All prices are from MilliporeSigma as of October 2024. Monomers are at least 99% purity and typically contain monomethyl ether hydroquinone as an inhibitor.

Name	Chemical specie	$/Liter
Butyl Acrylate (BA)	Monoacrylate	$65.60
2-Ethylhexyl acrylate (EHA)	Monoacrylate	$52.50
2-hydroxyethyl methacrylate (2-HEMA)	Monomethacrylate	$54.40
Methyl methacrylate (MMA)	Monomethacrylate	$70
Hydroxypropyl methacrylate (HPMA)	Monomethacrylate	$206
Poly(ethylene glycol) diacrylate (PEGDA)	Diacrylate	$587.00
1,4-butanediol diacrylate (BDDA)	Diacrylate	$306.00
Diethylene glycol diacrylate (DEGDA)	Diacrylate	$935.00
Urethane acrylate methacrylate (UDMA)	Dimethacrylate	$5770
Pentaerythritol triacrylate (PETA)	Triacrylates	$1010.00
Pentaerythritol tetraacrylate	Tetracrylates	$627.00
Diethylene glycol divinyl ether (DEGDE)	Vinyl ethers	$3740.00
1,4-Cyclohexanedimethanoldivinyl ether (CHDMDE)	Vinyl ethers	$956.00
Triethylene glycol divinyl ether(TEGDE)	Vinyl ethers	$271.00
Styrene	-	$57.00
N-vinyl pyrrolidone (NVP)	-	$213.20
3,4-Epoxycyclohexylmethyl-3′,4′-epoxycyclohexane carboxylate	Epoxides	$976.00
Diclycidylether derivative ofbisphenol A (ADE)	Epoxides	$175.60

Table S2. Formulations of soft, stretchable elastomeric resins for VP printing.

The resin consists of spacer (BA), sticker (EAEA), and crosslinker (BDDA) monomers, which can form a network under ultraviolet triggered free radical polymerization with the help from a photoinitiator (BAPO).

Sample Name	BA (mmol)	BDDA (mmol)	EAEA (mmol)	BAPOs (mmol)	Avobenzone (wt.%)	x	y	z	Crosslinker (%)
S0.3	19.5	0.029	-	0.039	0.1	0.375	0	249.25	0.3
S0.5	19.5	0.049	-	0.039	0.1	0.625	0	248.75	0.5
S0.75	19.5	0.074	-	0.039	0.1	0.937	0	248.12	0.75
S1.0	19.5	0.098	-	0.039	0.1	1.250	0	247.50	1
S2.5	19.5	0.25	-	0.040	0.1	3.125	0	243.75	2.5
S0.5 + 49% sticker	9.8	0.049	9.7	0.039	0.1	0.625	123.75	125	0.5

Table S3. Cost of commercially available elastomeric resins for VP printing.

Commercial resins are significantly more expensive than the commodity acrylate, butyl acrylate, which is the majority component of the resins.

Manufacturer	Name	Young's modulus or Shore A hardness	Tensile Breaking Strain	Cost	Source
Formlabs	Elastic 50A	N/A<br>50A	160%	199$ per liter	[2]
Formlabs	Silicone 40A	N/A<br>40A	230%	349$ per Liter	[3]
Carbon	EPU 40	8 MPa<br>68A	300%	~250$ per L, *estimated. Requires printer subscription to purchase*	[4]
Carbon	SIL30	N/A<br>35A	350%	~250$ per L, *estimated, Requires printer subscription to purchase*	[5]
Spot-A Materials	Elastic	N/A<br>65A	65%	64$ per L	[6]
3D Systems	Rubber-65A	23 MPa<br>65A	126%	249$ per Kg	[7]
Liqcreate	Flexible-X	N/A<br>55A	160%	140$ per Kg	[8]
3Dresyns	Bioflex A10 MF	<1 MPa<br>10A	>300%	385$ per Kg	[9]
resione	F39T	2 MPA<br>60A	255%	75$ per Kg	[10]
Adaptive3D	Soft ToughRubber	N/A<br>28.6 A	255%	175$ per Kg	[11]
Adaptive3D	Elastic ToughRubber 70	N/A<br>70A	400%	175$ per Kg	[12]
Henkel	IND402	42 MPa<br>76A	230%	300$ per Kg	[13]

Table S4. UV curing properties.

Gelation time decreases and cured storage modulus increases with increasing crosslinker and sticker concentrations.

Formulation	Gelation time (s)	Storage modulus after being cured (kPa)	Loss tan δ of the cured resin
S2.5	4.06 ± 0.69	184.21 ± 0	0.02 ± 0.00
S1.0	7.68 ± 0.10	65.5 ± 10.8	0.09 ± 0.01
S0.75	9.90 ± 0.14	44.8 ± 1.3	0.17 ± 0.01
S0.5	14.17 ± 0.51	35.5 ± 1.6	0.30 ± 0.01
S0.3	23.88 ± 0.04	21.9 ± 0.9	0.52 ± 0.01
S0.2	30.52 ± 9.66	18.4 ± 0.4	0.67 ± 0.10
S0.5 + 49% Sticker	4.04 ± 0.05	122.0 ± 21.2	0.42 ± 0.05

Table S5. Tensile properties of molded elastomers.

Elastomers possess an exceptional combination of softness and stretchability.

Formulation	Stress at break (kPa)	Strain at break (%)	Young's modulus (kPa)	Tensile toughness (kJ/m^3)
S2.5	285.2 ± 26.8	177.6 ± 25.2	237.7 ± 11.9	2.8 ± 0.6
S0.75	228.8 ± 82.1	542.9 ± 261.6	102.3 ± 22.8	8.0 ± 5.8
S0.5	164.8 ± 26.4	714.3 ± 220.2	73.9 ± 9.7	7.2 ± 3.2
S0.3	129.9 ± 17.8	891.8 ± 203.7	48.4 ± 7.7	9.3 ± 1.6
S0.5 + 49% sticker	279.1 ± 53.1	572.8 ± 56.8	120.2 ± 13.6	9.4 ± 2.5

Table S6. Tensile properties 3D printed elastomers.

Resins with crosslinker concentration lower than 0.75% introduces a new range of softness with strain at break for photocurable and 3D printable materials.

Formulation	Stress at break (kPa)	Strain at break (%)	Young's modulus (kPa)	Tensile toughness (kJ/m^3)
S2.5	199.2 ± 18.8	76.4 ± 18.4	278.8 ± 64.6	0.8 ± 0.3
S0.75	244.4 ± 26.1	514.1 ± 87.8	80.0 ± 11.1	6.9 ± 1.9
S0.5	182.5 ± 28.1	665.9 ± 109.0	55.8 ± 2.1	6.7 ± 2.2
S0.3	116.4 ± 39.5	1355.6 ± 249.4	20.3 ± 10.3	7.2 ± 2.8
S0.5 + 49% sticker	397.0 ± 80.0	646.2 ± 201.0	143.0 ± 18.3	14.5 ± 6.3

Table S7. Batch-to-batch comparison of 0.5% crosslinker samples.

	Batch #1	Batch #2	P-value Comparison
**Strain**	665.90 ± 109.01	628.87 ± 85.97	0.5852
**Stress**	182.49 ± 28.05	131.54 ± 36.69	0.0564
**Modulus**	55.81 ± 2.07	25.70 ± 7.78	0.0001
**Number of Samples:**	4	5	

Table S8. List of data points in Figure 2F.

Mechanical properties of existing VP printable materials.

Chemistry	Color/Symbol	Young's Modulus (Pa)	Tensile Breaking Strain	Reference
Thiol-ene	Dark Green	83,000	1.10	Wallin et al. (2017)[14]
		56,000	1.11	
		19,000	1.85	
		6,000	4.27	
		223,000	0.48	
		287,000	0.54	
		85,000	0.76	
		32,000	1.51	
		9,000	3.48	
Thiol-ene and silicone condensation dual network	Light Green	670,000	1.8	Wallin et al. (2022)[15]
		560,000	2.4	
		220,000	2.8	
		100,000	4.11	
Methacrylate	Pink	937,000	0.70	Bhattacharjee et al. (2018)[16]
		750,000	0.95	
		680,000	1.10	
		600,000	1.25	
		550,000	1.50	
		520,000	1.60	
Acrylate	Light Blue	7,660,000	10.45	Patel et al. (2017)[17]
		7,110,000	10.34	
		4,640,000	9.95	
		2,810,000	8.53	
		1,400,000	4.98	
		1,040,000	3.49	
		700,000	2.52	
		420,000	1.90	
Diels-Alder and acrylate	Orange	740,000	2.46	Durand-Silva et al. (2021)[18]
		1,190,000	1.91	
		3,580,000	1.28	
Acrylate and ionic liquid	Dark Blue	273,420	5.04	He et al. (2022)[19]
		284,850	5.84	
		302,960	7.23	
		328,760	8.32	
		384,780	9.70	

Table S9. Compression properties of our 3D printed resins.

Resins with crosslinker concentration lower than 2.5% can sustain up to 80% compression.

Formulation	Compressive Modulus (kPa)	Toughness at 80% (kJ/m^3)
S2.5	841.7 ± 41.7	0.4 ± 0.0
S0.75	257.5 ± 19.3	1.5 ± 0.1
S0.5	209.3 ± 11.9	1.5 ± 0.3
S0.3	88.1 ± 0.0	0.3 ± 0.0
S0.5 + 49% sticker	602.9 ± 3.9	3.5 ± 0.5

Table S10. Characteristics of 3D printed structures under impact.

The soft architected 3D structures decrease the severity of the impact by reducing maximum deformation, peak deceleration, and maximum voltage.

Condition	Maximum acceleration (g's)	Minimum acceleration (g's)	Maximum deformation (mm/mm)	Maximum voltage (V)	Modified head impact criteria
No protection	65.7 ± 6.2	-68.5 ± 8.5	0.35 ± 0.06	0.73 ± 0.35	1 ± 0.24
Bulk 0.5% crosslinker material	52.9 ± 5.7	-52.9 ± 7.6	0.29 ± 0.06	0.46 ± 0.09	0.42 ± 0.11
19.5% relative density	64.9 ± 12.7	-43.5 ± 5.9	0.26 ± 0.03	0.16 ± 0.08	0.59 ± 0.24
29.0% relative density	32.5 ± 6.0	-35.2 ± 3.6	0.29 ± 0.03	0.34 ± 0.05	0.25 ± 0.11
35.3% relative density	46.8 ± 5.1	-41.4 ± 3.9	0.24 ± 0.01	0.44 ± 0.03	0.35 ± 0.10

References

1 C. Mendes-Felipe, J. Oliveira, I. Etxebarria, J. L. Vilas-Vilela and S. Lanceros-Mendez, *Adv. Mater. Technol.*, 2019, **4**, 1800618.

2 Formlabs, Elastic 50A Resin 1 L, https://formlabs.com/store/materials/elastic-resin/.

3 Formlabs, Silicone 40A Resin, https://formlabs.com/store/materials/silicone-40a-resin/.

4 I. Carbon, EPU 40, https://www.carbon3d.com/materials/epu-40.

5 I. Carbon, SIL 30, https://www.carbon3d.com/materials/sil-30.

6 S.-A. Materials, Spot-E – Flexible, https://spotamaterials.com/wp/product/spot-e/.

7 3D-Systems, RUBBER-65A BLK, https://www.3dsystems.com/materials/figure-4-rubber-65a-blk.

8 Liqcreate, Flexible-X, https://www.liqcreate.com/product/flexible-x/.

9 3Dresyns, 3Dresyn Bioflex A10 MF Monomer Free, https://www.3dresyns.com/products/3dresyn-bioflex-a10-mf-monomer-free.

10 Resione, F39T Transparent Flexible Rubber-like 3D Printer Resin, https://www.resione.com/products/f39t.

11 Adaptive3D, Soft ToughRubber, https://adaptive3d.desktopmetal.com/additive-manufacturing/materials-used-for-additive-manufacturing/soft-toughrubber-2/.

12 Adaptive3D, Elastic ToughRubber, https://adaptive3d.desktopmetal.com/additive-manufacturing/materials-used-for-additive-manufacturing/elastic-toughrubber/.

13 Loctite, LOCTITE 3D IND402, https://www.loctiteam.com/ind402-high-rebound/.

14 T. J. Wallin, J. H. Pikul, S. Bodkhe, B. N. Peele, B. C. Mac Murray, D. Therriault, B. W. McEnerney, R. P. Dillon, E. P. Giannelis and R. F. Shepherd, *J. Mater. Chem. B*, 2017, **5**, 6249–6255.

15 T. J. Wallin, L. E. Simonsen, W. Pan, K. Wang, E. Giannelis, R. F. Shepherd and Y. Mengüç, *Nat. Commun.*, 2020, **11**, 1–10.

16 N. Bhattacharjee, C. Parra-Cabrera, Y. T. Kim, A. P. Kuo and A. Folch, *Adv. Mater.*, 2018, **30**, 1800001.

17 D. K. Patel, A. H. Sakhaei, M. Layani, B. Zhang, Q. Ge and S. Magdassi, *Adv. Mater.*, 2017, **29**, 1–7.

18 A. Durand-Silva, K. P. Cortés-Guzmán, R. M. Johnson, S. D. Perera, S. D. Diwakara and R. A. Smaldone, *ACS Macro Lett.*, 2021, **10**, 486–491.

19 X. He, J. Cheng, Z. Li, H. Ye, X. Wei, H. Li, R. Wang, Y. F. Zhang, H. Y. Yang, C. Guo and Q. Ge, *ACS Appl. Mater. Interfaces*, DOI:10.1021/acsami.2c18954.
